# UCP3 reciprocally controls CD4+ Th17 and Treg cell differentiation

**DOI:** 10.1371/journal.pone.0239713

**Published:** 2020-11-19

**Authors:** Emma B. O’Connor, Natalia Muñoz-Wolf, Gemma Leon, Ed C. Lavelle, Kingston H. G. Mills, Patrick T. Walsh, Richard K. Porter

**Affiliations:** 1 School of Biochemistry and Immunology, Trinity Biomedical Sciences Institute, Trinity College Dublin, Dublin, Ireland; 2 Department of Clinical Medicine, School of Medicine, Trinity College Dublin, Dublin 2, Ireland and National Children’s Research Centre, Our Lady’s Children’s Hospital, Crumlin, Dublin, Ireland; Northumbria University, UNITED KINGDOM

## Abstract

Uncoupling proteins (UCPs) are members of the mitochondrial anion carrier superfamily that can mediate the transfer of protons into the mitochondrial matrix from the intermembrane space. We have previously reported UCP3 expression in thymocytes, mitochondria of total splenocytes and splenic lymphocytes. Here, we demonstrate that *Ucp3* is expressed in peripheral naive CD4^+^ T cells at the mRNA level before being markedly downregulated following activation. Non-polarized, activated T cells (Th0 cells) from *Ucp3*^-/-^ mice produced significantly more IL-2, had increased expression of CD25 and CD69 and were more proliferative than *Ucp3*^+/+^ Th0 cells. The altered IL-2 expression observed between T cells from *Ucp3*^+/+^ and *Ucp3*^-/-^ mice may be a factor in determining differentiation into Th17 or induced regulatory (iTreg) cells. When compared to *Ucp3*^+/+^, CD4^+^ T cells from *Ucp3*^-/-^ mice had increased FoxP3 expression under iTreg conditions. Conversely, *Ucp3*^-/-^ CD4^+^ T cells produced a significantly lower concentration of IL-17A under Th17 cell-inducing conditions *in vitro*. These effects were mirrored in antigen-specific T cells from mice immunized with KLH and CT. Interestingly, the altered responses of *Ucp3*^-/-^ T cells were partially reversed upon neutralisation of IL-2. Together, these data indicate that UCP3 acts to restrict the activation of naive T cells, acting as a rheostat to dampen signals following TCR and CD28 co-receptor ligation, thereby limiting early activation responses. The observation that *Ucp3* ablation alters the Th17:Treg cell balance *in vivo* as well as *in vitro* suggests that UCP3 is a potential target for the treatment of Th17 cell-mediated autoimmune diseases.

## Introduction

Since the discovery of uncoupling protein 1 (UCP1) in brown adipose tissue (BAT) in the 1970s [1], other UCPs of the mitochondrial inner membrane have been reported. UCP2 [2, 3] and UCP3 [4, 5] were discovered in 1997, while UCP4 [6] and UCP5, also known as brain-specific mitochondrial carrier protein 1 (BMCP1; [7]), were discovered shortly afterwards. UCP3 has an amino acid sequence more similar to that of the archetypal UCP, UCP1, than any other member of the mitochondrial anion carrier superfamily (excluding UCP2), at 57% homology [4, 8, 9]. Its degree of homology to mitochondrial carriers other than UCP1 and UCP2 is approximately 30% in comparison [2, 10, 11]. The six membrane-spanning α-helix structure of UCP1 is highly conserved in UCP3, including the three mitochondrial carrier protein motifs, consistent with a role as an ion transporter of the mitochondrial inner membrane [2, 4]. Thus, it followed naturally that, upon discovery, this protein was assumed to have uncoupling activity similar to that of UCP1. However, while UCP3 is expressed in BAT [12], no role in non-shivering thermogenesis is apparent [13, 14], as none of the expected phenotypes of cold sensitivity or obesity are observed in *Ucp3*^-/-^ mice [11, 15]. Nevertheless, uncoupling activity of UCP3 has been demonstrated in mitochondria isolated from skeletal muscle of 3,4-methylenedioxymethamphetamine (MDMA)-treated rats and mice [16]. Since its discovery in skeletal muscle [4], UCP3 protein has been reported in BAT [12], spleen, thymus, reticulocytes, monocytes, lymphocytes [17], pancreatic β-cells [18] and heart [14].

We have previously reported that UCP3 is expressed in murine thymocytes, splenocyte mitochondria and splenic lymphocytes [17, 19]. The ablation of *Ucp3* has been reported to influence the frequency of CD4^+^ and CD8^+^ T cells in the thymus and spleen by affecting the apoptotic potential of these cells [19]. Together with the finding of UCP3 expression in thymocytes and the lymphocyte fraction of the spleen, this suggests that UCP3 is important in thymic function and may therefore have a role in T cell selection, maturation, and/or function. However, the role of UCP3 in peripheral T cell responses has not been thoroughly investigated. Upon antigen encounter in the periphery, CD4^+^ T cells can undergo a process of differentiation towards defined effector Th subsets to carry out specific functions. Th1 cells produce IFN-γ and mediate cellular immune responses against intracellular pathogens, in particular viruses but also intracellular bacteria [20, 21]. Th17 cells promote neutrophil recruitment and antimicrobial peptide production and mediate protective immunity against fungi and extracellular bacteria at sites of infection [20, 22, 23]. In contrast, Treg cells that express the signature transcription factor FoxP3 [21, 24, 25] act to suppress effector Th1 and Th17 responses and thereby maintain immune system homeostasis and prevent the development of autoimmunity [21, 24, 26]. The objective of this study was to determine whether UCP3 plays a role in peripheral CD4^+^ T cell activation and differentiation towards different effector Th subsets.

## Materials and methods

### Mice

Mice were maintained in compliance with the Health Products Regulatory Authority regulations and with the approval of the University of Dublin’s ethical review board. The members of the Ethics Committee are: Dr. Jean Fletcher (Chair), Dr. Emma Creagh (Director of Research), Dr. Sarah Doyle (External Advisor), Dr. Frederick Sheedy and Dr. Andreea Petrasca. Experiments were performed under license from the Health Products Regulatory Authority (license numbers: AE19136/P020 and AE19136/P088) and in strict accordance with regulations laid out by Laboratory Animal Science and Training–Ireland and the European Union [(Protection of Animals Used for Scientific Purposes)Regulations 2012 (S.I. number 543 of 2012) and Directive 2010/63/EU].

*Ucp3*^-/-^ mice were provided to our laboratory by Prof. Pádraic Fallon [Trinity Biomedical Sciences Institute, Trinity College Dublin; JAX^®^ strain number 005937]. *Ucp3*^-/-^ mice were of a mixed genetic background of C57BL/6J and 129S strains and wildtype littermates of *Ucp3*^-/-^ mice were used as controls. *Ucp3*^+/+^ and *Ucp3*^-/-^ mice were bred in-house in the Comparative Medicine Unit, Trinity Biomedical Sciences Institute, Trinity College Dublin and were housed in individually ventilated cages in a specific pathogen-free facility at room temperature. A standard 12 h light/dark cycle was in place and animals had access to H_2_O and standard laboratory rodent chow *ad libitum*. At the time of sacrifice, mice were euthanized by CO_2_ asphyxiation followed by cervical dislocation. For all experiments, *Ucp3*^-/-^ mice and *Ucp3*^+/+^ controls were age- and sex-matched.

### Reagents

APC:anti-mouse CD44, CellTrace^™^ Violet Cell Proliferation Kit, DPBS, eFluor^™^ 660:anti-mouse CD25, FBS, FITC:anti-mouse CD62L, FoxP3/Transcription Factor Staining Buffer Set, High Capacity cDNA Reverse Transcription Kit, IL-2 monoclonal antibody (clone JES6-1A12), L-glutamine, LIVE/DEAD^™^ Fixable Aqua Dead Cell Stain Kit, PE:anti-mouse IL-2, PE:anti-mouse/rat FoxP3, Pen Strep, PerCP-Cy^™^5.5:anti-mouse CD69, RPMI, TaqMan^®^ Gene Expression Assays, TaqMan^®^ Universal PCR Master Mix, TrackIt^™^ Cyan/Yellow Loading Buffer and UltraPure^™^ DNase/RNase-Free dH_2_O were purchased from Bio-Sciences Ltd. Anti-mouse CD3ε, anti-mouse CD28, anti-mouse IFN-γ, anti-mouse IL-4, APC:anti-mouse IFN-γ, IFN-γ, IL-17A and IL-2 ELISA kits, ISOLATE II RNA Mini Kit, Pacific Blue^™^:anti-mouse CD69, PE:anti-mouse IL-17A, PE-Cy7:anti-mouse CD4, recombinant IL-6 (rIL-6) and rIL-12 were purchased from Medical Supply Co. Ltd. 2-mercaptoethanol, acetic acid, agarose, antimycin A, brefeldin A, D-(+)-glucose, EDTA, FCCP, ionomycin, Nancy-520, oligomycin, PMA, Red Blood Cell Lysing Buffer Hybri-Max^™^, rotenone, RPMI 1640 medium with L-glutamine, without glucose and sodium bicarbonate, sodium bicarbonate and Trizma^®^ Base were purchased from Sigma-Aldrich. CD4 Microbeads (mouse) were purchased from Miltenyi Biotec Ltd. Recombinant human TGF-β1 (rhTGF-β1) was from ImmunoTools GmbH. Corning^®^ Cell-Tak Cell and Tissue Adhesive and GeneRuler^™^ DNA Ladder Mix were from Fischer Scientific Ltd. The Seahorse XF24 FluxPak was purchased from Agilent Technologies. Keyhole limpet haemocyanin (KLH) was from Merck Millipore and cholera toxin (CT) was purchased from List Biological Laboratories, Inc. Complete RPMI (cRPMI) was made up of RPMI supplemented with 10% (w/v) FBS, 2 mM L-glutamine, 1% (w/v) Pen Strep and 0.0003% 2-mercaptoethanol. MACS buffer was made up of DPBS supplemented with 2% (w/v) FBS and 2 mM EDTA. TAE buffer was made up of 40 mM Trizma^®^ Base, 1 mM acetic acid, 1 mM EDTA and dH_2_O.

### CD4^+^ T cell isolation and culture

Spleen cells were isolated according to the method of Mills [27]. Following splenocyte isolation, CD4^+^ T cells were isolated from the splenocyte suspension by positive selection using CD4 Microbeads, LS columns and the MidiMACS Starting Kit according to the manufacturer’s instructions (Miltenyi Biotec Ltd.). 0.2 x 10^6^ CD4^+^ T cells were stimulated *in vitro* with the indicated concentrations of anti-CD3 and/or anti-CD28 and the appropriate cytokine mix for up to 120 h at 37°C and 5% CO_2_.

### Quantitative RT-PCR

Total RNA isolation from 1 x 10^6^ CD4^+^ T cells was carried out using the ISOLATE II RNA Mini Kit as per the manufacturer’s instructions and quantified using a NanoDrop^®^ ND-1000 Spectrophotometer (Thermo Fisher Scientific, Waltham, MA, U.S.A.) and ND-1000 software (version 3.7.1). The High Capacity cDNA Reverse Transcription Kit was used to reverse transcribe isolated RNA into cDNA according to the manufacturer’s instructions. Following reverse transcription, TaqMan^®^ Gene Expression Assays for *Ucp3* and *Hprt* (ID numbers: Mm00494077_m1 and Mm00446968_m1, respectively) and TaqMan^®^ Universal PCR Master Mix were used to perform PCR as per the manufacturer’s guidelines. The relative expression level of each mRNA was calculated using the comparative C_T_ method described by Schmittgen and Livak [28]. Following PCR, samples were electrophoresed on 2.5% (w/v) agarose TAE gels. Nancy-520-stained DNA bands were photographed using a Kodak^®^ GEL Logic 200 Imaging System (Carestream Health Inc., Rochester, NY, U.S.A.) and Kodak^®^ ID Image Analysis software (version 3.6; Eastman Kodak Company, Rochester, NY, U.S.A.).

### Flow cytometry

For intracellular cytokine analysis, 1 x 10^6^ CD4^+^ T cells were stimulated with 10 ng.mL^-1^ of PMA, 1 μg.mL^-1^ of ionomycin and 5 μg.mL^-1^ of brefeldin A for 6 h at 37°C with 5% CO_2_. After this time, samples were washed and stained with LIVE/DEAD^™^ Fixable Aqua Dead Cell Stain, PE-Cy7:anti-CD4, eFluor^™^ 660:anti-CD25 and/or Pacific Blue^™^:anti-CD69 as per the manufacturers’ instructions. Samples were fixed and permeabilised using the FoxP3/Transcription Factor Staining Buffer Set before being stained with PE:anti-IL-2, APC:anti-IFN-γ, PE:anti-IL-17A or PE:anti-FoxP3 following the manufacturers’ guidelines. Cells were washed and resuspended in 0.25 mL of DPBS. Flow cytometry was carried out using a BD FACSCanto^™^ II or BD LSRFortessa^™^ (BD Biosciences, CA, U.S.A.). Samples were processed using BD FACSDiva^™^ software (version 8.0; BD, Franklin Lakes, NJ, U.S.A.).

### Proliferation assay

Following CD4^+^ T cell isolation, cells were resuspended in 5 mL of warm DPBS containing 1 μM CellTrace^™^ Violet and incubated at 37°C for 10 min. 10 mL of cRPMI were added to each tube to stop the labelling process and samples were incubated on ice for 15 min. Cells were washed, resuspended in cRPMI and cultured as described previously.

### ELISA

Cell supernatants were taken at designated time points, transferred to 1.5 mL tubes and stored at -20°C until ready to use. IFN-γ, IL-17A and IL-2 ELISAs were carried out according to the manufacturer’s instructions (BioLegend^®^). Supernatant samples were diluted with ELISA assay diluent at 1:2, 1:5, 1:10 and/or 1:20 dilutions. Following completion of the assay, the absorbance of each well was measured at 450 nm using a SpectraMax Plus 384 microplate reader (Molecular Devices, San Jose, CA, U.S.A.) and SoftMax Pro software (version 6.4.2; Molecular Devices). Concentration values were multiplied by the appropriate dilution factor.

### Immunisation of *Ucp3*^+/+^ and *Ucp3*^-/-^ mice

*Ucp3*^+/+^ and *Ucp3*^-/-^ mice were immunized subcutaneously with 1 μg of KLH alone or 1 μg of KLH plus 1 μg of CT in a 200 μL volume of sterile DPBS (100 μL in each flank) on days 0 and 14. On day 21, splenocytes were isolated from immunized mice and analysed by flow cytometry for FoxP3 expression. For *ex vivo* antigen-specific recall response analysis, the remaining splenocytes were diluted to a density of 2 x 10^6^ cells per mL, plated in 200 μL volumes (0.4 x 10^6^ cells per well) and incubated in the presence of cRPMI alone or with 2 or 50 μg.mL^-1^ of KLH. After 72 h, supernatants were collected to analyse cytokine secretion by ELISA.

### Statistical analysis

Data are presented as mean ± SEM unless otherwise indicated. Flow cytometry data were analysed using FlowJo^®^ software (version 10.0.7; FlowJo^®^ LLC, Ashland, Oregon, U.S.A.). All other data were analysed using GraphPad Prism^®^ software for Windows (version 5; GraphPad Software, Inc., La Jolla, CA, U.S.A.). Statistical significance was detected and quantified using a two-tailed, unpaired *t* test or a one-way or two-way, repeated measures or non-repeated measures ANOVA with a *post hoc* Bonferroni or Dunnett test, where indicated. A *p* value of < 0.05 was used to indicate significance where detected. Experiments were performed three times unless otherwise stated.

## Results

### *Ucp3* transcription is downregulated in activated CD4^+^ T cells

Following the discovery by our laboratory of UCP3 expression in thymocytes and splenic lymphocytes [17, 19], we examined the expression of *Ucp3* in peripheral CD4^+^ T cells using quantitative RT-PCR. This was performed on primary CD4^+^ T cells following isolation and on activated, non-polarized T cells (Th0 cells) 24, 48 and 72 h post-stimulation to determine the relative changes in expression as cells undergo activation.

**Fig 1:**
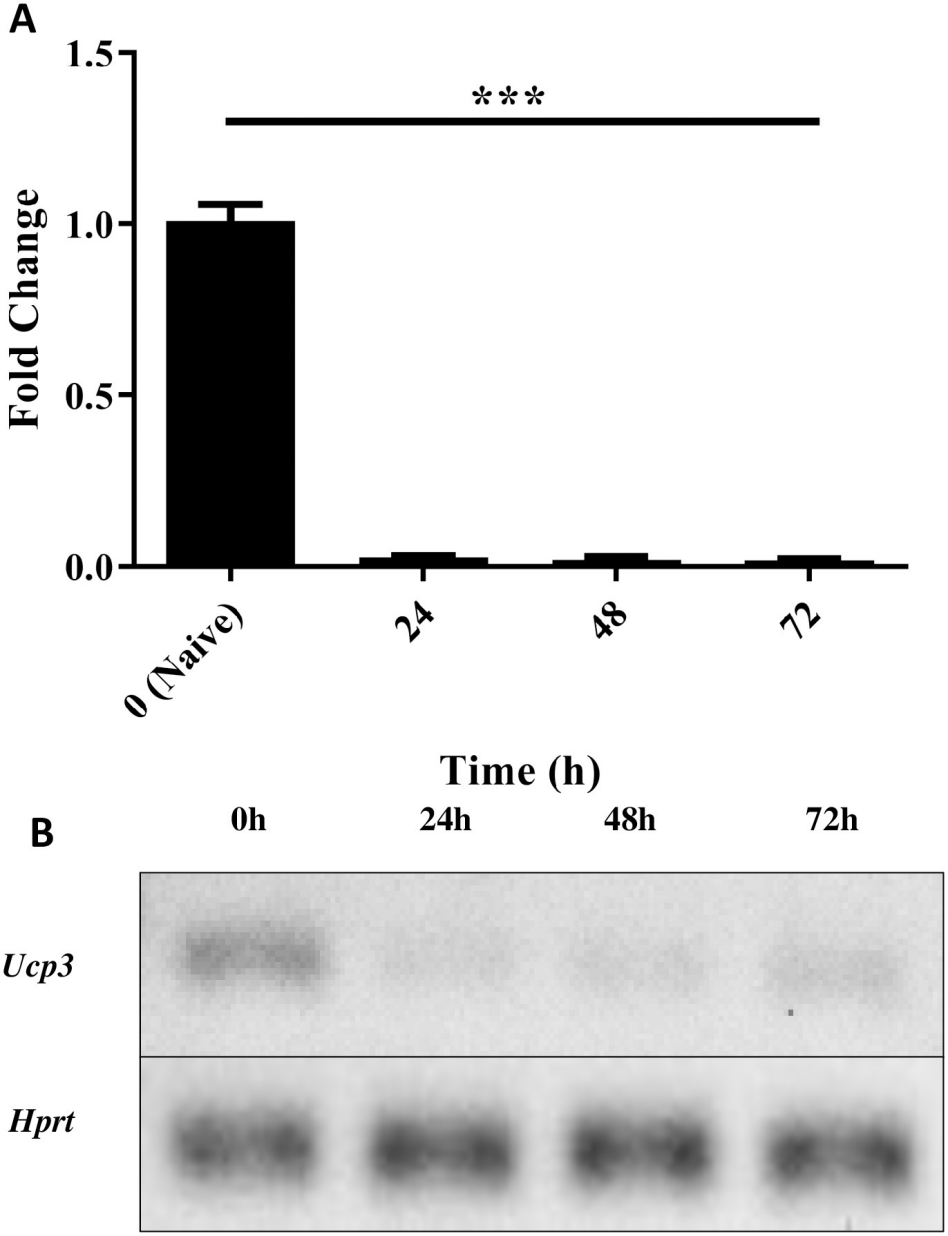
*Ucp3* gene expression is downregulated in Th0 cells. Primary CD4^+^ T cells were isolated from a suspension of splenocytes and analysed by RT-PCR immediately or after activation under non polarising conditions (Th0) as described in Methods. **(A)** RT-PCR analysis of *Ucp3* gene expression in Th0 cells relative to naive T cells. RT-PCR was performed three times in triplicate. Data were analysed using a one-way repeated measures ANOVA with a *post hoc* Dunnet test to quantify significance where detected. *** = *p* < 0.001. **(B)** Representative agarose gel of naive T cell cDNA and Th0 cell cDNA prepared at the times indicated, displaying *Ucp3* and *Hprt* at 69 and 83 bp, respectively.

Using the comparative C_T_ method to calculate relative gene expression, *Ucp3* cDNA converted from mRNA was first normalised to *Hprt* as an endogenous control to account for variability in the initial concentration and quality of the total RNA and in the conversion efficiency of the reverse transcription reaction. Gene expression was then measured by the quantitation of normalised *Ucp3* cDNA relative to that of the naive T cell sample. *Ucp3* expression is significantly decreased in Th0 cells within 24 h of stimulation compared to naive T cells and remains at a significantly lower level up to 72 h post-stimulation (Fig 1A). Fig 1B displays a representative agarose gel for *Ucp3* in naive T cell and Th0 cell cDNA from the RT-PCR experiments. Our data suggest that UCP3 is expressed in peripheral naive CD4^+^ T cells before being rapidly downregulated in response to T cell activation. This suggests that UCP3 may have an important role in naive CD4^+^ T cell function in addition to its perceived role in thymic function and T cell selection.

#### *Ucp3* ablation does not affect Th1 cells but reciprocally affects Th17 and iTreg cell function and generation, respectively

**Fig 2:**
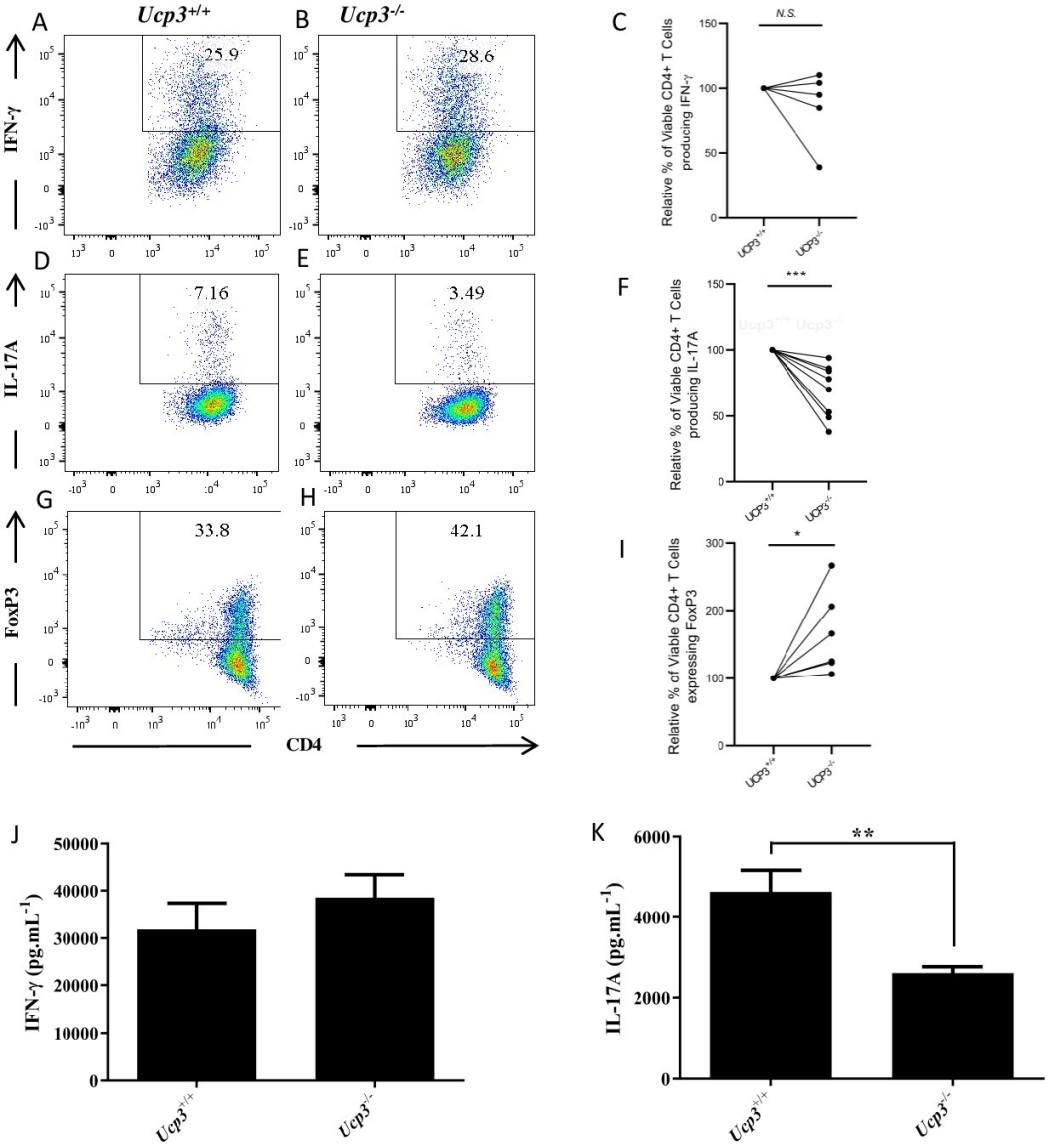
*Ucp3* ablation has no effect on Th1 cells but reciprocally affects Th17 and iTreg cell generation. Purified CD4^+^ T cells from *Ucp3*^+/+^ and *Ucp3*^-/-^ mice were differentiated to specific effector subsets as described in Methods. IFN-γ production by Th1 cells 72 h post-stimulation, was measured by flow cytometry and ELISA. **(A&B)** Representative FACS data and **(C)** collated FACS data from 6 independent experiments for IFN-γ expression by *Ucp3*^+/+^ and *Ucp3*^-/-^ Th1 cells. **(D&E)** Representative FACS data and **(F)** collated FACS data from 6 independent experiments for IL-17A expression by *Ucp3*^+/+^ and *Ucp3*^-/-^ Th17 cells. **(G&H)** Representative FACS plots and **(I)** collated data for FoxP3 expression by *Ucp3*^+/+^ and *Ucp3*^-/-^ iTreg cells. Analysis of levels of secretion of **(K)** IFN-γ by Th1 cells and (L) IL-17A by Th17 cells from *Ucp3*^+/+^ and *Ucp3*^-/-^ mice. ELISAs were performed at least three times in sextuplicate. ELISA and FACS expression data were analysed using a two-tailed, unpaired *t* test (* = p < 0.05; ** = p < 0.01; *** = p<0.001).

To investigate the potential significance of *Ucp3* downregulation upon CD4^+^ T cell activation, we examined whether UCP3 plays a role in Th1, Th17 and iTreg differentiation. Firstly, we compared cytokine production by Th1 and Th17 cells from *Ucp3*^+/+^ compared with *Ucp3*^-/-^ mice. Importantly, basal expression of the activation marker, CD69, was unchanged in *Ucp3*^-/-^ CD4^+^ T cells (S1A Fig). Similarly, no differences in the relative proportion of memory and natural CD4 regulatory T (nTreg) cells were detected in the spleens of *Ucp3*^-/-^ mice (S1B and S1C Fig, respectively), demonstrating that *Ucp3* deficiency does not significantly alter the homeostasis of peripheral CD4^+^ T cell subsets.We found no significant difference in IFN-γ production by Th1 cells detected by flow cytometry (Fig 2A, 2B and 2C) or by ELISA (Fig 2J). The viability of *Ucp3*^+/+^ and *Ucp3*^-/-^ Th1 cells was not significantly different (S2A and S2B Fig). In contrast, the expression of IL-17A by Th17 cells from *Ucp3*^-/-^ mice was significantly decreased compared with Th17 cells isolated from wildtype littermate controls, as determined by flow cytometry (Fig 2D, 2E and 2F) and ELISA (Fig 2K). In addition, the viability of *Ucp3*^-/-^ Th17 cells was lower than that of *Ucp3*^+/+^ Th17 cells (S2C and S2D Fig). Furthermore, there was a significantly increased percentage of FoxP3^+^ iTreg cells generated following differentiation of *Ucp3*^-/-^ CD4^+^ T cells under iTreg-polarizing conditions (Fig 2G, 2H and 2I) and these cells displayed greater viability when compared with their wildtype counterparts (S2E and S2F Fig). These data demonstrate that, while *Ucp3* ablation does not affect Th1 differentiation, it has a clear impact on the generation of Th17 and iTreg cells *in vitro*.

### *Ucp3* ablation affects Th0 cell activation

**Fig 3:**
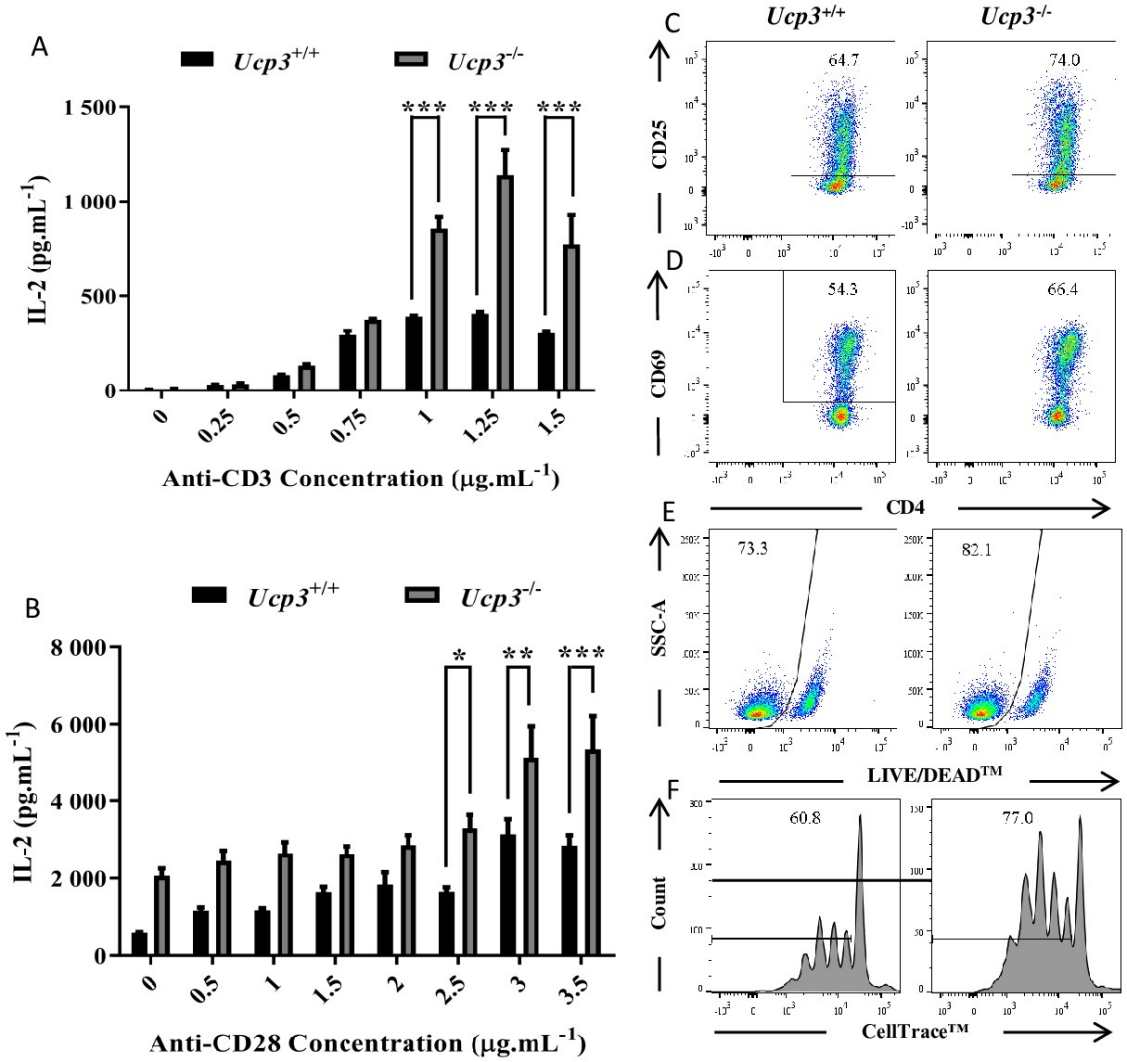
Primary activation of *Ucp3*^-/-^ Th0 cells is altered compared to that of *Ucp3*^+/+^ Th0 cells. Purified naive CD4^+^ T cells isolated from *Ucp3*^+/+^ and *Ucp3*^-/-^ mice were activated in the presence of increasing concentrations of anti-CD3 **(A)**, increasing concentrations of anti-CD28 in the presence of 1 μg.mL^-1^ of anti-CD3 **(B)**. Levels of IL-2 secretion by Th0 cells after 24 h activation as determined by ELISA of Th0 cells. Data were analysed using a two-way ANOVA with a *post hoc* Bonferroni test to quantify significance where detected. * = *p* < 0.05. ** = *p* < 0.01. *** = *p* < 0.001. FACS analysis of **(C)** CD25 and **(D)** CD69 expression on Th0 cells at 24 h, and **(E)** viability at 24 h and **(F)** proliferation at 72 h post activation with 0.25 μg.mL^-1^ of anti-CD3. Dot plots and histograms are representative of at least three different experiments. Data were analysed using a two-tailed, unpaired *t* test to quantify significance where detected (* = *p* < 0.05). Flow cytometry data are presented as a percentage of the viable, CD4^+^ T cell population **(C, D, F, G)** or the total CD4^+^ T cell population **(E)**.

To explore how *Ucp3* ablation may be altering Th17 and iTreg cell differentiation and given that *Ucp3* expression is decreased early after CD4^+^ T cell activation, we investigated the effect of *Ucp3* ablation on the early activation responses of CD4^+^ T cells. IL-2 has been extensively characterized as an effector cytokine secreted by activated CD4^+^ T cells in response to appropriate antigen presentation and co-stimulation through the TCR and CD28 co-receptor [21, 24, 29]. As *Ucp3* gene expression is significantly decreased upon activation (Fig 1), we next explored the effect of *Ucp3* ablation on IL-2 expression 24 h post-stimulation by ELISA. IL-2 secretion by *Ucp3*^-/-^ Th cells was significantly greater compared with wildtype cells in the presence of 1, 1.25 and 1.5 μg.mL^-1^ of anti-CD3 alone (Fig 3A). This increase in expression was also observed upon co-stimulation with titrated amounts of anti-CD28 in the presence of 1 μg.mL^-1^ of anti-CD3 (Fig 3B). These data indicate that UCP3 may play a role in restricting IL-2 expression in response to TCR stimulation in the absence and presence of CD28-mediated co-stimulation.

In order to further investigate these effects, we analysed the expression of CD25 early after T cell activation. CD25 is the α-subunit present in the receptor for IL-2 [24] and is absent or minimally expressed on resting T cells, with its expression potently induced following stimulation via the TCR or contact with IL-2 itself [21, 24]. As the greatest differences in IL-2 secretion were observed upon stimulation with anti-CD3 alone (Fig 3A), we analysed CD25 expression under these conditions. Similar to IL-2, CD25 was expressed at higher levels in activated *Ucp3*^-/-^ CD4^+^ T cells (Fig 3C). Together, these data suggest that, in the absence of *Ucp3* expression, CD4^+^ T cells are more sensitive to activation stimuli. In further support of these observations, the expression of CD69, an early T cell activation marker, was significantly increased (Fig 3D).

As IL-2 is a T cell growth factor that promotes T cell survival and proliferation [21, 24, 29], we analysed activated CD4^+^ T cell viability and expansion post-stimulation. These data revealed that, in association with increased IL-2 levels, *Ucp3*^-/-^ CD4^+^ T cells displayed increased survival after 24 h and proliferation after 72 h (Fig 3E and 3F, respectively). Together, these data demonstrate that, in the absence of *Ucp3*, peripheral CD4^+^ T cells have a lower threshold for TCR-mediated activation *in vitro*, pointing towards a role for UCP3 in restricting TCR signalling and early CD4^+^ T cell activation responses.

### The increased production of IL-2 by *Ucp3*^-/-^ T cells impacts the differentiation of Th17 and iTreg cells

**Fig 4:**
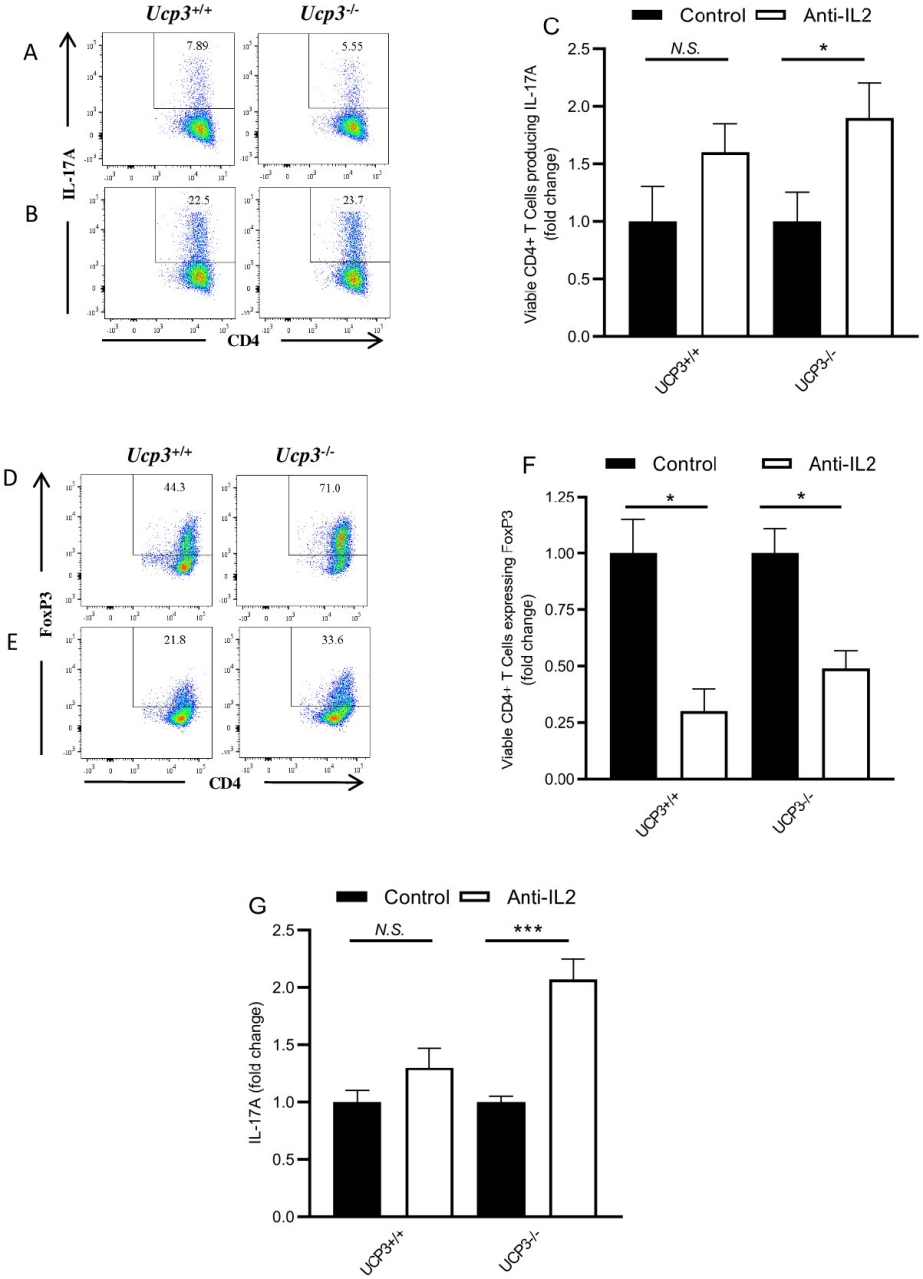
IL-2 neutralisation restores *Ucp3*^-/-^ T_H_17 and iTreg cells to a *Ucp3*^+/+^ phenotype. *Ucp3*^+/+^ and *Ucp3*^-/-^ T_H_17 were differentiated in the absence **(A)** or presence **(B)** of 10 μg.mL^-1^ of anti-IL-2. Relative levels of IL-17A expression by T_H_17 cells 72 h post-stimulation were measured by flow cytometry **(A, B,** with collated data **C)** and ELISA **(G)**. Levels of FoxP3 expression in iTreg cells generated in the absence **(D)** or presence **(E)** of 10 μg.mL^-1^ of anti-IL-2 are shown together with collated data **(F)**. Data shown are representative of five independent experiments with similar results. Data were analysed using a student’s t-test to quantify significance where detected (* = *p* < 0.05; ** = *p* < 0.01; ** = *p* < 0.001; *N*.*S*. = no significance).

IL-2 can play a significant role in influencing Th17 and Treg cell differentiation. High levels of IL-2 in the presence of high levels of TGF-β can promote the induction and survival of iTreg cells and the expression of FoxP3 and inhibit Th17 cell polarization, while lower levels of TGF-β in the presence of IL-6 favour Th17 cell polarization [21, 24, 30]. The significantly higher production of IL-2 protein by *Ucp3*^-/-^ Th0 cells suggests that an increase in IL-2 production may be one factor responsible for the effects observed on Th17 and iTreg cells. To test whether an increase in IL-2 production contributes to enhanced iTreg and decreased Th17 cell generation in the absence of *Ucp3*, we differentiated CD4^+^ T cells under these conditions in the presence and absence of an anti-IL-2 neutralising antibody. In agreement with previous reports [21, 24, 30–33], IL-2 neutralisation led to an 1.6-fold increase in IL-17A expression by wildtype Th17 cells as determined by FACS. While a similar effect was observed on *Ucp3*^-/-^ Th17 cells, it was notable that IL-2 neutralisation led to a 1.9-fold increase in IL-17A(Fig 4A, 4B and 4G; S5A, S5B, S5C and S5D Fig). As expected, IL-2 neutralisation had an opposing effect on iTreg cells, with decreased induction of FoxP3 expression, as determined by FACS, in both *Ucp3*^+/+^ (3.3-fold decrease) and *Ucp3*^-/-^ (2-fold decrease) CD4^+^ T cells (Fig 4D, 4E and 4F). In addition, and as predicted from the FACS data, IL-2 neutralization led to a 1.3-fold increase in IL17A protein released from Th17 cells from *Ucp3*^+/+^ mice and a 2-fold increase in IL-17A production from *Ucp3*^-/-^ mice, as determined by ELISA (Fig 4G; S5E Fig). Note we were able to demonstrate that addition of the isotypic antibody to the anti-IL-2 neutralising antibody was equivalent to adding no antibody to Th17 cell and did not increase IL17A production (S5 Fig). It is noteworthy that the addition of anti-IL-2 neutralizing antibody has a more pronounced effect on Th17 differentiation in UCP3 knock CD4+ T cells when compared to their wild type counterparts. The inhibitory effects on iTreg differentiation are similarly significant irrespective of UCP3 expression. These data suggest that increased IL-2 secretion as a result of *Ucp3* ablation may be a contributing factor to increased iTreg cell differentiation and diminished Th17 responses. Interestingly, in a comparison of cells from wildtype and UCP 3-/- mice, there was no significant difference in IL-2 transcript levels in non-polarised Th0 cells (S3 Fig) suggesting a differential effect on translation between cells from *Ucp3*^+/+^ and *Ucp3*^-/-^. In a further attempt to examine a mechanistic link between UCP3 and the fate of T cell Th17 and Treg cells, we were aware from the literature that reactive oxygen species (ROS) have been implicated in IL-2 production by Tcells [34], however we saw no difference in reactive oxygen species (ROS) production in Th0 cells from UCP3 knockout and wildtype mice (S4 Fig). We also looked at oxygen consumption rate (OCR) data (index of oxidative metabolism) and extracellular acidification rate (ECAR) data (index of glycolytic flux). Analysis of the data demonstrated a reduced OCR/ECAR in Th0 cells from UCP3 knockout animals when compared to those from wildtypes (S6 Fig). Araujo *et al*. [35] demonstrated that reducing OCR/ECAR of cultured T cells results in reduced Th17 and increased Treg cells. Thus one might postulate that the reduced OCR/ECAR that we see in Th0 cells, due to the absence of UCP3, may result in reduced Th17 and increased Treg cells. One interpretation of these data is that UCP3 may not be affecting mitochondrial efficiency *per se*, but may well be redirecting substrate supply to mitochondria as has been suggested in several publications [14, 36, 37] *e*.*g*. by facilitating fatty acid transport and driving a greater OCR/ECAR ratio.

### *Ucp3* ablation alters CD4^+^ T cell antigen recall response and Treg cell frequency *in vivo*

We have shown that *Ucp3* ablation alters the Th17:Treg cell ratio *in vitro* by decreasing the differentiation of Th17 cells and enhancing the generation of FoxP3^+^ iTreg cells. Furthermore, these effects result at least in part through increased IL-2 production by *Ucp3*^-/-^ cells. To investigate whether the effect of *Ucp3* ablation on activated CD4^+^ T cells is mirrored in the *in vivo* setting and to further explore the role of UCP3 in CD4^+^ T cell function, we examined the effect of *Ucp3* ablation on CD4^+^ T cell responses *in vivo* following immunization with the model T cell antigen, keyhole limpet haemocyanin (KLH), in the presence or absence of the adjuvant, cholera toxin (CT).

CT is a potent mucosal immunogen and adjuvant used experimentally to promote cellular immune responses to co-administered antigens, enhancing the induction of CD4^+^ Th cell and cytotoxic T lymphocyte responses [38]. We specifically chose this approach as recent reports have described the potent Th17 cell-driving activity of CT [22, 23]. *Ucp3*^-/-^ and littermate control mice were immunized on days 0 and 14. On day 21 after immunization, splenocytes were isolated and analysed *ex vivo* for FoxP3 expression by flow cytometry. Additionally, T cell antigen-specific recall responses were analysed by culturing splenocytes in the presence of increasing doses of KLH (2 or 50 μg.mL^-1^) for 72 h followed by analysis of Th-derived cytokine expression in the supernatants by ELISA.

**Fig 5:**
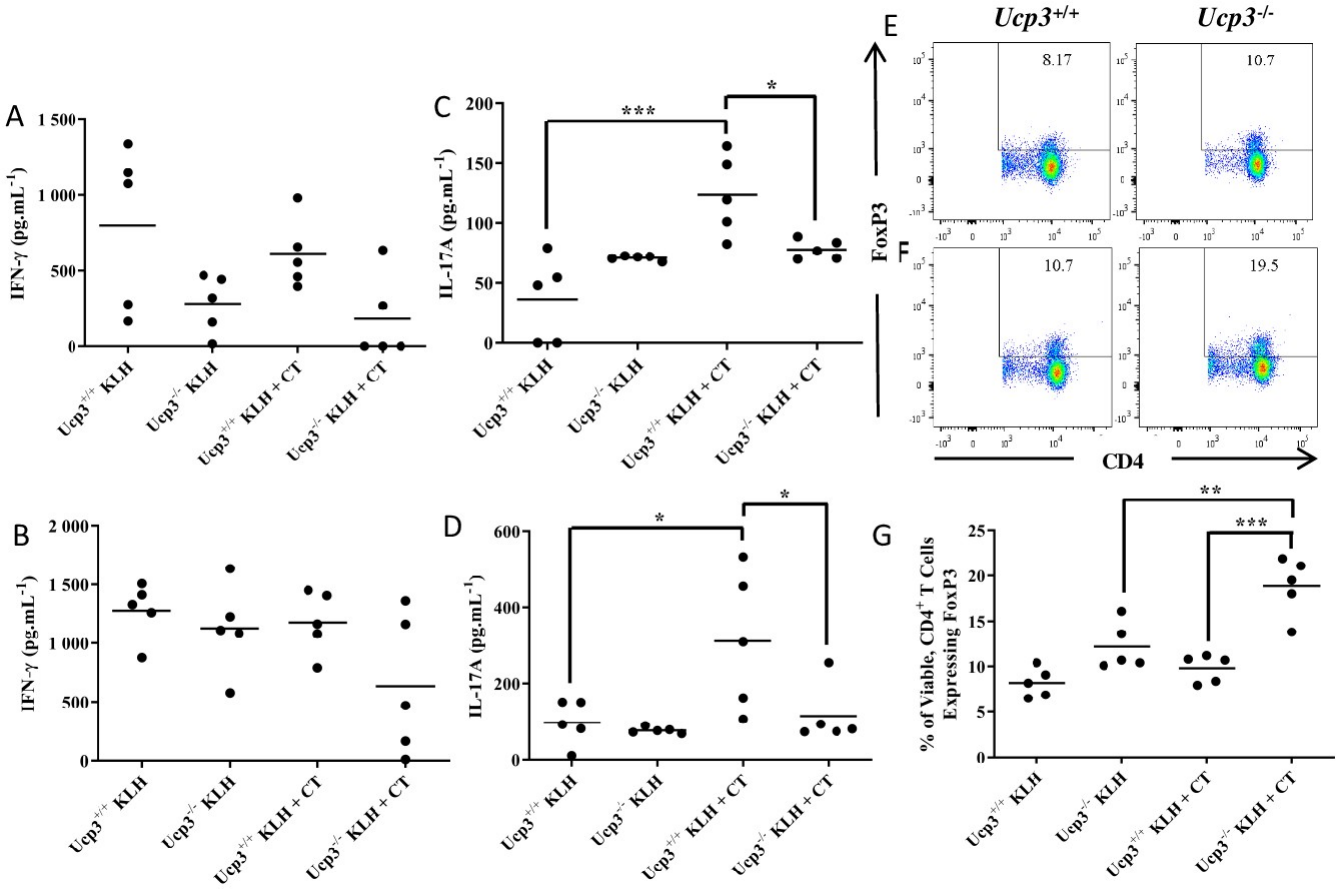
*In vitro* effects of *Ucp3* ablation on Th17 and iTreg cells are mirrored *in vivo*. *Ucp3*^+/+^ and *Ucp3*^-/-^ mice were immunized with 1 μg of KLH or 1 μg of KLH and CT on day 0 and re-immunized on day 14. On day 21, splenocytes were isolated from immunized mice and incubated in the presence of 2 **(A, C)** or 50 **(B, D)** μg.mL^-1^ of KLH for 72 h before supernatants were taken for ELISAs. IFN-γ **(A, B)** and IL-17A **(C, D)** secretion, as measured by ELISA. ELISAs were performed once in triplicate. Data are presented as mean cytokine concentration. Alternatively, on day 21, splenocytes were immediately analysed for FoxP3^+^ iTreg frequency. **(E–G)** Frequency of CD4^+^FoxP3^+^ Treg cells in mice immunized with KLH only **(E)** or KLH and CT **(F)**. All data are representative of a single experiment with 5 mice per goup as indicated. Data were analysed using a two-way ANOVA with a *post hoc* Bonferroni test to quantify significance where detected. * = *p* < 0.05. ** = *p* < 0.01. *** = *p* < 0.001.

Although there seemed to be a trend for less IFN-γ secretion by spleen cells from *Ucp3*^-/-^ mice after 72 h compared with the corresponding cells from *Ucp3*^+/+^ mice (Fig 5A and 5B), this was not statistically significant, reflecting the *in vitro* data (Fig 2A, 2B, 2C and 2J). As CT is known to suppress Th1 responses following immunization, even in the presence of a Th1-promoting antigen such as LPS [39, 40], a lack of enhanced secretion of IFN-γ following immunization with KLH plus CT compared to KLH alone is perhaps unsurprising. In contrast to IFN-γ, marked differences in the secretion of IL-17A were observed upon *ex vivo* restimulation of spleen cells with antigen. As expected, spleen cells from *Ucp3*^+/+^ mice immunized with KLH plus CT secreted significantly more IL-17A compared with cells from mice immunized with KLH alone when restimulated with 2 or 50 μg.mL^-1^ of KLH (Fig 5C and 5D, respectively). Interestingly, these differences were not observed in *Ucp3*^-/-^ splenocytes. Additionally, IL-17A secretion by spleen cells from *Ucp3*^-/-^ mice was significantly less than spleen cells from *Ucp3*^+/+^ mice immunized with KLH and CT. These data directly mirror our *in vitro* findings that *Ucp3*^-/-^ Th17 cells produce significantly less IL-17A than their wildtype counterparts (Fig 2D, 2E, 2F and 2K). Similarly, significant differences were observed in the frequency of CD25^+^FoxP3^+^ iTreg cells. The frequency of splenic CD25^+^FoxP3^+^ iTreg cells in *Ucp3*^-/-^ mice immunized with antigen plus adjuvant (KLH plus CT) was significantly higher than that observed in *Ucp3*^+/+^ mice, as well as *Ucp3*^-/-^ mice immunized with antigen alone (Fig 5E–5G). This is consistent with our *in vitro* observations of enhanced FoxP3^+^ iTreg cell generation from CD4^+^ T cells isolated from UCP3-deficient mice (Fig 2G, 2H and 2I). The above data confirm that *Ucp3* ablation alters CD4^+^ T cell function *in vivo* in a manner similar to that observed *in vitro*.

## Discussion

Our studies demonstrate that UCP3 can play a profound role in directing the activation responses of CD4^+^ T cells. We have shown that *Ucp3* ablation affects early CD4^+^ T cell activation through significantly higher IL-2 production and secretion under a number of different stimulatory conditions. In line with this finding, *Ucp3*^-/-^ CD4^+^ T cells displayed elevated expression of CD25, the α subunit of the IL-2R. These data indicate that UCP3 plays a role in restricting activation and IL-2 expression in response to TCR and CD28 co-stimulation. In agreement with this, *Ucp3*^-/-^ Th0 cells displayed increased upregulation of the early activation marker CD69, higher cell viability and higher frequencies of cells that have undergone more cell divisions. It is interesting to note that, in a similar manner, CD4^+^ lymphocytes of *Ucp2*^-/-^ mice produce higher levels of IL-2 and TNF-α than wildtype controls [41] and Pecqueur *et al*. [42] reported that loss of *Ucp2* activated cell proliferation specifically in murine embryonic fibroblasts and T cells. It is clear from our work that *Ucp3*^-/-^ cells undergo earlier activation compared to their wildtype counterparts. Intriguingly, no effect on *Ucp3*^-/-^ Th1 cell viability or production of IFN-γ, the characteristic Th1 cytokine, was detected. Considering the effect of *Ucp3* ablation on CD4^+^ T cell activation, these results are somewhat surprising as one may have predicted an increase in survival of and IFN-γ production by *Ucp3*^-/-^ Th1 cells due to the increased IL-2 production observed in *Ucp3*^-/-^ cells. IL-2 promotes Th1 cell differentiation by inducing IL-12Rβ2 subunit expression via STAT5 (augmenting responsiveness to IL-12) and is capable of driving IFN-γ production [21, 24]. It is unclear as of yet why *Ucp3*^-/-^ Th1 cell activation, function and viability are unaffected by *Ucp3* ablation, in contrast to their non-polarized counterparts, as well as under Th17- and iTreg-inducing conditions.

Importantly, *Ucp3* ablation perturbs CD4^+^ T cell function *in vivo*, mirroring our data generated through *in vitro* experiments. *Ucp3*^-/-^ Th17 cells display significantly less IL-17A production than *Ucp3*^+/+^ Th17 cells. The viability of *Ucp3*^-/-^ Th17 cells is also decreased, which may account at least in part for the decreased IL-17A production observed; however, IL-2 can act to constrain IL-17A production by promoting STAT5 signalling, with STAT5 binding to the *Il-17* gene locus and inhibiting *Il-17* transcription [30–33]. FoxP3^+^ *Ucp3*^-/-^ cells are higher in number than FoxP3^+^ *Ucp3*^+/+^ cells and display higher cell viability. These data indicate that, while *Ucp3*^-/-^ Th1 cells are unaffected by the altered IL-2 production following *Ucp3* ablation, the generation of Th17 and Treg cells is crucially affected. These data are in agreement with several reports demonstrating the reciprocal role of IL-2 on Th17 and iTreg cell generation [30–33]. Exposure of activated CD4^+^ T cells to IL-2 leads to lower cell surface expression of IL-6Rβ which, together with IL-6Rα, forms the IL-6R [24]. Additionally, as mentioned above, IL-2 activates STAT5 signalling which inhibits the binding of STAT3 to the *Il-17a* locus by competing for the same sites on the gene; therefore, IL-2 reduces IL-6-mediated STAT3 activation which is required for the development of RORγt^+^ Th17 cells and the balance between STAT3 and STAT5 signalling is responsible for determining the extent of Th17 cell generation [24]. Conditions in which IL-2 or its receptor subunits are absent result in a deficiency of Treg cells [29], leading to a lack of peripheral immune tolerance and systemic autoimmunity [24]. Additionally, where IL-2 deficiency is not absolute, Treg cell function is still abnormal [29]. Since neutralising IL-2 activity directed the *Ucp3*^-/-^ CD4^+^ T cell responses to return to a more wildtype-like state, IL-2 may play a role in mediating the effects observed on *Ucp3*^-/-^ Th17 and iTreg cells.

In this study, we have demonstrated for the first time that *Ucp3* is rapidly downregulated in mature CD4^+^ T cells at the mRNA level following TCR and CD28 co-receptor stimulation and that *Ucp3* ablation significantly alters the function of non-polarized and polarized CD4^+^ T cells. Our data point towards a role for UCP3 in restricting TCR signalling and CD4^+^ T cell activation. UCP3 may act as a rheostat to inhibit the magnitude of TCR activation and fine-tune the TCR and CD28 signal, hence its expression in naive CD4^+^ T cells and subsequent downregulation after cell stimulation. Following activation, TCR signals may block or switch off *Ucp3* gene expression within 24 h in order to negate the dampening effect of UCP3 and allow for full activation. Ablation of *Ucp3* allows for heightened early activation, altering TCR signalling pathways and causing augmented IL-2 production, not through enhanced ROS production. Another possibility is that UCP3 may be influencing Th17/Treg ratio by facilitating an oxidative phenotype in Th0 cells.. Furthermore, the lack of differential levels of IL-2 transcripts in Th0 cells from wildtype and UCP3^-/-^ mice, may indicate that UCP3 manifests its effect at the level of IL-2 protein translation. Importantly, the augmented production of IL-2 by *Ucp3*^-/-^ activated T cells may perturb and reciprocally affect the generation of Th17 and iTreg cells.

Th17 cells are a pro-inflammatory subset implicated as playing a central role in many autoimmune disorders [20, 21]. Treg cells, on the other hand, play a unique and important role in preventing autoimmunity. They function in contrast to the other T cell subsets, modulating inflammation and acting as key players in sustaining peripheral self-tolerance. This helps to prevent excessive tissue damage from too much inflammation and autoimmune responses to self-antigen, as documented by the occurrence of autoimmunity due to Treg deficiencies [21, 25]. In the absence of IL-2 signals, Treg cell number declines substantially, whereas Th17 cell number increases, leading to an enhanced susceptibility to autoimmune disease and inflammatory disorders [24]. Our data demonstrate that, in the absence of UCP3, the opposite is occurring: increased IL-2 signals are promoting the generation of immunosuppressive Treg cells and decreasing the number of Th17 cells. Consequently, it could be postulated that this may lead to enhanced protection from the development and/or progression of autoimmune disease and inflammatory disorders, implicating *Ucp3* as a potential target for preventing or ameliorating Th17 cell-mediated autoimmune diseases such as psoriasis and multiple sclerosis. In light of these data, the targeting of *Ucp3* to modulate autoimmunity warrants further investigation.

## Supporting information

S1 File Supporting data for Fig 1A. Fold Change in UCP3 mRNA as a function of time in naïve/Th0 cells; Full gel images for Fig 1B in the manuscript. Three RT-PCR experiments were performed for the data in Fig 1. The products from all three experiments were ran on one gel (the image attached). The top half of the gel contains bands for *Hprt* and the bottom half contains bands for *Ucp3*. Experimental replicate #3 (the HPRT and UCP3 bands on right hand side of gel) were used to make Fig 1B in the manuscript. The samples on the gel are in the following order: 1. Ladder; 2. 0 h naive cells (taken ex-vivo); 3. TH0 cells following 24 h stimulated with 1 and 2 ug/mL of anti-CD3 and anti-CD28; 4. TH0 cells following 48 h stimulated with 1 and 2 ug/mL of anti-CD3 and anti-CD28; 5. TH0 cells following 72 h stimulated with 1 and 2 ug/mL of anti-CD3 and anti-CD28.
(ZIP)

S2 File Supporting data for Fig 2C, 2F, 2I, 2J and 2K. (ZIP)

S3 File Supporting data for Fig 3A and 3B. (ZIP)

S4 File Supporting data for Fig 4C, 4F ang 4G. (ZIP)

S5 File Supporting data for Fig 5A–5D and 5G. (ZIP)

S6 File Supporting data for S2B, S2D and S2F Fig. (ZIP)

S7 File Supporting data for S3 Fig. (PDF)

S8 File Supporting data for S4 Fig. (PDF)

S9 File Supporting data for S5D and S5E Fig. (ZIP)

S10 File Supporting data for S6 Fig. (PDF)

S1 Fig Basal CD4^+^ T cell expression of CD69 and relative proportions of memory and nTreg cells in *Ucp3*^+/+^ and *Ucp3*^-/-^ mice are comparable. Primary CD4^+^ T cells were isolated from *Ucp3*^+/+^ and *Ucp3*^-/-^ mice and the relative levels of early activated (CD69), naïve/memory CD4^+^ (CD44/CD62L), and Treg (FoxP3) subsets analysed. **(A)** CD69 expression of naive CD4^+^ T cells. **(B)** Frequency of naive and memory T cells. **(C)** FoxP3 and CD4 co-expression by CD4^+^ T cells. All data are presented as a percentage of the viable, CD4^+^ T cell population. Dot plots are representative of at least three different experiments.
(TIF)

S2 Fig Viability of Th1, Th17 and Treg cells from *Ucp3*^+/+^ and *Ucp3*^-/-^ mice in Fig 2. Differentiated Th subsets from *Ucp3*^+/+^ and *Ucp3*^-/-^ mice shown in Fig 2 were analysed for viability through staining with LIVE/DEAD^™^ exclusion. **(A&B)** Representative and collated viability data for *Ucp3*^+/+^ and *Ucp3*^-/-^ Th1 cells, **(C&D)** Th17 cells, **(E&F)** iTreg cells. Data are representative of six independent experiments. Data were analysed using a two-tailed, unpaired *t* test to quantify significance. Data are presented as a percentage of the total CD4^+^ T cell population.
(TIF)

S3 Fig *Il-2* gene expression is comparable in *Ucp3*^+/+^ and *Ucp3*^-/-^ Th0 cells. RT-PCR analysis of *Il-2* gene expression in Th0 cells relative to naive T cells. Primary CD4^+^ T cells were isolated from a suspension of splenocytes and analysed by RT-PCR immediately or after activation under non polarising conditions (Th0) as described in the methods section (conditions were 1 ug/mL of anti-CD3 and 2 ug/mL of anti-CD28). RT-PCR was performed three times in triplicate. Data were analysed using a two-way ANOVA with a *post hoc* Bonferroni test to quantify significance where detected.
(TIF)

S4 Fig Mitochondrial ROS production by naive CD4+ T cells from *Ucp3*+/+ and *Ucp3*-/- mice are comparable. Primary CD4+ T cells were isolated from a suspension of splenocytes and stained with DCFDA before being analysed on a flow cytometer. An example of the primary data is given and the histogram represents three different experiments detecting ROS in viable CD4^+^ T cell population. All data were analysed using a two-tailed, unpaired *t* test.
(TIF)

S5 Fig IL-2 neutralisation antibody increases T_H_17 cell phenotype when compared to (i) addition of isotype antibody or (ii) no addition of isotypic antibody UCP 3^+/+^ CD4+ Th cells magnetically purified from spleens of C57Bl/6 mice were differentiated towards a Th17 lineage with the addition of Th17 polarizing cytokines (20ng anti-IL4 + 20ng IL-6 + 10ug anti-IFN γ + 2.5ng TGFB) and cultured in the absence (A), or presence (B) of 10 μg.mL^-1^ of anti-IL-2 or (C) isotypic antibody. Collated data showing relative levels of IL-17a expression by T_H_17 cells 72h post-stimulation, were measured by flow cytometry (D) and ELISA (E). Data shown is representative of three independent experiments performed in triplicate. Data were analysed using a Students’ t-test to quantify significance where detected (* = p < 0.05; ** = *p* < 0.01; ** = p< 0.001; *N*.*S*. = no significance). The IL-2 Monoclonal Antibody (JES6-1A12) was from Invitrogen/Thermofisher (Catalog # 16-7022-81) and the isotypic antibody was the recommended isotypic antibody namely Rat IgG2a kappa Isotype Control (eBR2a), Invitrogen/Thermofischer (16-4321-82). Th17 polarzing cytokines (anti-IL4 (11B11, #14-7041-81), IL-6 (#RMIL6I), anti-IFNγ (XMG1.2, #BE0055), IL-17a ELISA kit (#14-7175-688) and FACs antibody (PE, eBio17B, #12-7177-81) are from eBioscience/Thermofisher. Recombinant human TGFB1 is from Immunotools, (#11343160).
(TIF)

S6 Fig Oxygen consumption rate/extracellular acidity ratio (OCR/ECAR ratio) for T_H_0 cells from *Ucp3*^+/+^ and *Ucp3*^-/-^ mice. T_H_0 cells were generated by incubating naive cells in the presence of 1 and 2 μg.mL^-1^ of anti-CD3 and anti-CD28, respectively, for 24h before being seeded at 500 000 cells per well in a Seahorse cell microplate and analysed on the Seahorse XF24 Analyzer. Low-buffered RPMI-1640 Medium (without sodium bicarbonate) with L-glutamine and 11.11mM glucose were used as the assay medium for the Seahorse OCR and ECAR analysis as per manufacturer’s guidelines. Seahorse experiments were performed three times in quintuplicate. Data are presented as mean ± SEM. Data were analysed using a two-tailed, unpaired *t* test to quantify significance where detected. A *p* value of < 0.05 was used to indicate significance. ** = *p* < 0.01.
(TIF)
