# Supplementary material for: UCP3 reciprocally controls CD4+ Th17 and Treg cell differentiation
Source: PLoS One. 2020 Nov 19;15(11):e0239713. doi: 10.1371/journal.pone.0239713 (PMC7676685; doi:10.1371/journal.pone.0239713)
Supplement: S1 File — Fold Change in UCP3 mRNA as a function of time in naïve/Th0 cells; Full gel images for Fig 1B in the manuscript. Three RT-PCR experiments were performed for the data in Fig 1. The products from all three experiments were ran on one gel (the image attached). The top half of the gel contains bands for Hprt and the bottom half contains bands for Ucp3. Experimental replicate #3 (the HPRT and UCP3 bands on right hand side of gel) were used to make Fig 1B in the manuscript. The samples on the gel are in the following order: 1. Ladder; 2. 0 h naive cells (taken ex-vivo); 3. TH0 cells following 24 h stimulated with 1 and 2 ug/mL of anti-CD3 and anti-CD28; 4. TH0 cells following 48 h stimulated with 1 and 2 ug/mL of anti-CD3 and anti-CD28; 5. TH0 cells following 72 h stimulated with 1 and 2 ug/mL of anti-CD3 and anti-CD28. (ZIP) [file pone.0239713.s001.zip › S1A_File.pdf]

0 (Naive)	24	48	72
0.924023	0.010697	0	0.010576
1.056286	0.015446	0	0.005208
1.024557	0.017595	0	0.005775
0.915734	0.033648	0.066831	0.025976
0.815072	0.029742	0.052398	0.009816
1.339784	0.050824	0.029313	0.023492
0.959929	0.016285	0.008625	0.042996
0.99103	0.027483	0.008262	0.022882
1.051173	0.037128	0.011595	0.019201
