# Supplementary material for: UCP3 reciprocally controls CD4+ Th17 and Treg cell differentiation
Source: PLoS One. 2020 Nov 19;15(11):e0239713. doi: 10.1371/journal.pone.0239713 (PMC7676685; doi:10.1371/journal.pone.0239713)
Supplement: S2 File — (ZIP) [file pone.0239713.s002.zip › S2C_File.pdf]

Ucp3 $^{+/+}$	*Ucp3* $^{-/-}$
100	85
100	110
100	95
100	39
100	104
100	110
