# Supplementary material for: UCP3 reciprocally controls CD4+ Th17 and Treg cell differentiation
Source: PLoS One. 2020 Nov 19;15(11):e0239713. doi: 10.1371/journal.pone.0239713 (PMC7676685; doi:10.1371/journal.pone.0239713)
Supplement: S2 File — (ZIP) [file pone.0239713.s002.zip › S2J_File.pdf]

$Ucp3^{+/+}$	$Ucp3^{-/-}$
21409.86	27794.17
21522.61	27490.99
15649.44	28212.61
44378.2	49815.4
42749.56	50742.48
45149.94	47214.58
