# Supplementary material for: UCP3 reciprocally controls CD4+ Th17 and Treg cell differentiation
Source: PLoS One. 2020 Nov 19;15(11):e0239713. doi: 10.1371/journal.pone.0239713 (PMC7676685; doi:10.1371/journal.pone.0239713)
Supplement: S2 File — (ZIP) [file pone.0239713.s002.zip › S2K_File.pdf]

$Ucp3^{+/+}$	$Ucp3^{-/-}$
1997.146	1788.954
2065.406	1840.46
2100.468	1829.91
4037.23	2912.5
4145.05	2796.145
4138.07	2766.67
5477.49	3083.75
5961.52	2920.86
5683.82	3206.31
6204.74	3232.34
7430.3	2726.6
6276.1	2376
