# Supplementary material for: UCP3 reciprocally controls CD4+ Th17 and Treg cell differentiation
Source: PLoS One. 2020 Nov 19;15(11):e0239713. doi: 10.1371/journal.pone.0239713 (PMC7676685; doi:10.1371/journal.pone.0239713)
Supplement: S3 File — (ZIP) [file pone.0239713.s003.zip › S3A_File.pdf]

					UCP3$^{+/+}$			
0.002	1.91		7.78	5.345	3.65		6.74	8.14
24.716	26.604	27.172	31.69	30.32	16.295	35.27	32	34.08
74.234	81.332	88.196	92.08	85.22	94.485	90.17	74.73	90.79
271.434	274.494	242.106	385.22	360	399.94	365.18	313.5	277.22
383.852	390.502	366.966	427.21	408.845	389.87	398.25	390.69	390.29
418.102	419.096	426.592	429.525	444.01	454.04	416.92	388.7	410.56
277.608	286.44	287.176	320.86	337.245	292.2	316.97	290.18	273.05

								UC[illegible]
						15.7		
	34.6	37.5	44.222	50.498	46.418	35.365	21.985	45.395
72.34	72.94	65.18	149.42	153.142	155.626	124.95	126.57	155.45
258.4	238.18	131.98	322.35	369.716	369.64	367.96	383.285	379.02
407.82	355.9	387.26	609.236	608.232	527.096	1041.43	1088.67	1021.74
368.3	378.2	329.4	579.364	701.438	733.162	1079.105	1014.665	1040.28
334.38	310.64	314.88	379.518	433.578	421.484	573.485	565.665	576.095

[illegible]3$^{-/-}$

4.52	8.14	3.69			
39.16	41.56	43.36	12.92		1.52
83.98	138.39	131.13	69.94	150.08	140.86
385.91	399.04	380.73	383.96	387.26	379.02
1000.15	892.65	764.63	1100.48	970.7	647.9
1141.13	1840.94	1952.89	1725.84	918.58	939.12
950.37	802.7	635.15	558.34	1062.8	2324.48
