# Supplementary material for: UCP3 reciprocally controls CD4+ Th17 and Treg cell differentiation
Source: PLoS One. 2020 Nov 19;15(11):e0239713. doi: 10.1371/journal.pone.0239713 (PMC7676685; doi:10.1371/journal.pone.0239713)
Supplement: S3 File — (ZIP) [file pone.0239713.s003.zip › S3B_File.pdf]

						$UCP3^{+/+}$		
0	604.158	609.096	642.196	610.235	623.77	578.81	608.49	575.82
0.5	890.336	825.958	800.766	1109	1137.86	993.69	1622.52	1454.77
1	932.784	902.782	880.142	1126.65	1117.695	1212.77	1363.77	1315.4
1.5	1057.158	1102.062	968.28	1470.65	1661.775	1229.16	2074.65	2329.96
2	1168.422	1111.63	1192.19	5111.845	1789.915	1279.41	1645.54	1687.4
2.5	1068.422	1104.53	1023.364	1569.505	1633.595	1433.57	2162.87	1919.82
3	1472.654	1416.206	1368.378	2688.49	2581.845	2699.505	3755.17	3978.08
3.5	1526.65	1516.272	1510.116	2632.975	2663.82	2673.07	3451.62	3349.04

561.76	600.76	531.76	439.84	1122.722	1129.148	1115.056	2301.1	2776.035
1368.47	1314.18	1240.6	1315.1	881.992	1144.706	1127.51	2513.915	2591.98
1323.38	1369.66	1158.9	1403.34	1101.154	1150.8	1184.676	2656.405	2755.385
2056.08	1891.12	1984.58	1884.88	1565.31	1566.702	1528.22	2634.02	2707.705
1622.28	1844.38	1792.94	1827.24	1537.184	1558.666	1548.62	2818.04	2802.61
1871.03	1898.9	1976.8	2106.02	1692.704	1469.044	1509.214	3565.255	3162.23
3427.54	4723.4	5504.92	4111	1672.538	1641.552	1621.838	3752.425	3669.325
3372.24	3932.1	3902.86	3692.3	1610.222	1610.222	1594.56	3770.89	3832.455

UCP3$^{-/-}$

2757.575	1743.36	2433.6	2355.7	2524.06	1824.74	2663.64
2475.345	3048.64	2677	2895.91	3504.04	3358.94	3215.44
2405.08	3300.08	3348.86	2986.72	3716.68	3754.52	3450.16
2572.685	3309.65	3120.48	3084.96	3047.58	3213.54	3246.12
2802.22	3504.19	3492.61	3347.48	3793.18	3734.32	3449.2
3352.905	4371.56	4641.58	3329.73	5198.02	3525.14	3754.46
3864.12	6324.86	6591.31	6039.87	7414.3	9163.98	9750.46
3843.45	6844.52	6681.87	6444.46	9916.38	9190.5	8684.6
