# Supplementary material for: UCP3 reciprocally controls CD4+ Th17 and Treg cell differentiation
Source: PLoS One. 2020 Nov 19;15(11):e0239713. doi: 10.1371/journal.pone.0239713 (PMC7676685; doi:10.1371/journal.pone.0239713)
Supplement: S4 File — (ZIP) [file pone.0239713.s004.zip › S4C_File.pdf]

S4C File. Supporting data for Figure 4C fold change FACS data

UCP3+/+ IL17A	UCP3+/+Anti IL2	UCP3-/- IL17A	UCP3-/-Anti IL2
1.87	2.28	1.65	2.15
0.74	1.08	0.47	1.08
0.53	1.68	0.77	1.91
0.87	1.53	1.12	2.49
