# Supplementary material for: UCP3 reciprocally controls CD4+ Th17 and Treg cell differentiation
Source: PLoS One. 2020 Nov 19;15(11):e0239713. doi: 10.1371/journal.pone.0239713 (PMC7676685; doi:10.1371/journal.pone.0239713)
Supplement: S4 File — (ZIP) [file pone.0239713.s004.zip › S4F_File.pdf]

S4F File. Supporting data for Figure 4F fold change FACS data

UCP3+/+ FoxP3	UCP3+/+ FoxP3 Anti IL2	UCP3-/- FoxP3	UCP3-/- FoxP3 Anti IL2
0.64	0.32	0.93	0.62
1.14	0.56	1.29	0.61
0.87	0.06	0.76	0.3
1.35	0.26	1.02	0.43
