# Supplementary material for: UCP3 reciprocally controls CD4+ Th17 and Treg cell differentiation
Source: PLoS One. 2020 Nov 19;15(11):e0239713. doi: 10.1371/journal.pone.0239713 (PMC7676685; doi:10.1371/journal.pone.0239713)
Supplement: S4 File — (ZIP) [file pone.0239713.s004.zip › S4G_File.pdf]

S4G File. Supporting data for Figure 4G fold change ELISA data

UCP3+/+ IL-17A	UCP3+/+ IL-17A Anti IL2	UCP3-/- IL-17A	UCP3-/- IL17A Anti IL2
0.77	0.95	0.92	1.64
0.8	0.95	0.94	1.67
0.82	0.93	0.97	1.72
1.37	1.62	1.17	2.43
1.08	1.62	0.89	2.34
1.16	1.81	1.11	2.62
