# Supplementary material for: UCP3 reciprocally controls CD4+ Th17 and Treg cell differentiation
Source: PLoS One. 2020 Nov 19;15(11):e0239713. doi: 10.1371/journal.pone.0239713 (PMC7676685; doi:10.1371/journal.pone.0239713)
Supplement: S5 File — (ZIP) [file pone.0239713.s005.zip › S5A_File.pdf]

Ucp3$^{+/+}$ KLH	Ucp3$^{-/-}$ KLH	p3$^{+/+}$ KLH +	p3$^{-/-}$ KLH + CT
1146.486	15.46233	460.6757	631.8276
1073.76	468.878	653.4813	0
1339.947	443.615	981.2397	0
164.7417	320.6923	397.4643	269.7293
278.314	158.071	554.8915	0
