# Supplementary material for: UCP3 reciprocally controls CD4+ Th17 and Treg cell differentiation
Source: PLoS One. 2020 Nov 19;15(11):e0239713. doi: 10.1371/journal.pone.0239713 (PMC7676685; doi:10.1371/journal.pone.0239713)
Supplement: S5 File — (ZIP) [file pone.0239713.s005.zip › S5B_File.pdf]

Ucp3$^{+/+}$ KLH	Ucp3$^{-/-}$ KLH	p3$^{+/+}$ KLH +	p3$^{-/-}$ KLH + CT
1411.908	576.7087	1163.819	1360.945
1329.995	1635.553	1080.103	165.0697
1507.381	1225.992	1451.606	10.97833
875.268	1085.407	789.2	1163.109
1260.441	1110.505	1405.127	469.589
