# Supplementary material for: UCP3 reciprocally controls CD4+ Th17 and Treg cell differentiation
Source: PLoS One. 2020 Nov 19;15(11):e0239713. doi: 10.1371/journal.pone.0239713 (PMC7676685; doi:10.1371/journal.pone.0239713)
Supplement: S5 File — (ZIP) [file pone.0239713.s005.zip › S5C_File.pdf]

Ucp3$^{+/+}$ KL	Ucp3$^{-/-}$ KLH	p3$^{+/+}$ KLH +	p3$^{-/-}$ KLH + CT
0	72.09333	164.0203	88.762
0	67.60033	82.607	76.766
48.056	71.77866	148.9687	69.93667
54.5035	70.341	119.27	70.61066
79.282	72.58733	101.028	83.68533
