# Supplementary material for: UCP3 reciprocally controls CD4+ Th17 and Treg cell differentiation
Source: PLoS One. 2020 Nov 19;15(11):e0239713. doi: 10.1371/journal.pone.0239713 (PMC7676685; doi:10.1371/journal.pone.0239713)
Supplement: S5 File — (ZIP) [file pone.0239713.s005.zip › S5D_File.pdf]

Ucp3$^{+/+}$ KLH	Ucp3$^{-/-}$ KLH	p3$^{+/+}$ KLH +	p3$^{-/-}$ KLH + CT
151.3053	76.36134	533.167	254.1057
10.584	72.093	107.139	80.54
150.9907	68.00433	455.393	74.25
81.798	78.563	311.0767	73.03667
92.94067	89.84067	162.448	93.92934
