# Supplementary material for: UCP3 reciprocally controls CD4+ Th17 and Treg cell differentiation
Source: PLoS One. 2020 Nov 19;15(11):e0239713. doi: 10.1371/journal.pone.0239713 (PMC7676685; doi:10.1371/journal.pone.0239713)
Supplement: S5 File — (ZIP) [file pone.0239713.s005.zip › S5G_File.pdf]

$Ucp3^{+/+}$ KLH	$Ucp3^{-/-}$ KLH	$Ucp3^{+/+}$ KLH + CT	$Ucp3^{-/-}$ KLH + CT
6.87	10.1	7.95	13.8
6.49	10.4	8.39	18
8.17	10.7	10.7	21.1
9.08	16.1	10.8	19.5
10.4	13.6	11.2	21.9
