# Supplementary material for: UCP3 reciprocally controls CD4+ Th17 and Treg cell differentiation
Source: PLoS One. 2020 Nov 19;15(11):e0239713. doi: 10.1371/journal.pone.0239713 (PMC7676685; doi:10.1371/journal.pone.0239713)
Supplement: S6 File — (ZIP) [file pone.0239713.s006.zip › SS6B_File.pdf]

$Ucp3^{+/+}$	$Ucp3^{-/-}$
48.1	53.93
54.8	66.95
39.25	27.95
81	76.6
43.15	47.7
75.9	80.5
