# Supplementary material for: UCP3 reciprocally controls CD4+ Th17 and Treg cell differentiation
Source: PLoS One. 2020 Nov 19;15(11):e0239713. doi: 10.1371/journal.pone.0239713 (PMC7676685; doi:10.1371/journal.pone.0239713)
Supplement: S6 File — (ZIP) [file pone.0239713.s006.zip › SS6D_File.pdf]

$Ucp3^{+/+}$	$Ucp3^{-/-}$
62.3	45.03
68	58.53
72.25	45.2
83.2	69.4
64.15	47.25
96.9	91.2
