# Supplementary material for: UCP3 reciprocally controls CD4+ Th17 and Treg cell differentiation
Source: PLoS One. 2020 Nov 19;15(11):e0239713. doi: 10.1371/journal.pone.0239713 (PMC7676685; doi:10.1371/journal.pone.0239713)
Supplement: S6 File — (ZIP) [file pone.0239713.s006.zip › SS6F_File.pdf]

$Ucp3^{+/+}$	$Ucp3^{-/-}$
32.8	46.45
67.05	78.65
41.9	62.9
34.2	35.7
33.2	47.8
48.1	77.5
28.6	32.7
43	59.5
41.1	45.8
42.6	50.6
