# Supplementary material for: UCP3 reciprocally controls CD4+ Th17 and Treg cell differentiation
Source: PLoS One. 2020 Nov 19;15(11):e0239713. doi: 10.1371/journal.pone.0239713 (PMC7676685; doi:10.1371/journal.pone.0239713)
Supplement: S7 File — (PDF) [file pone.0239713.s007.pdf]

$Ucp3^{+/+}$

0 (Naive)	1.097332	1.025267	0.888843	1.036701	0.970635	0.993781	1.085982	0.984184
4	45.87597	40.24302	39.11545	31.3125	25.66387	27.83188	110.5585	97.25272
8	43.25115	43.31115	36.90301	21.50102	21.42663	20.63946	107.2128	107.585
12	32.04439	26.77844	26.28194	13.70218	15.18246	14.41335	66.88759	58.51225
16	23.04955	18.69612	21.88191	9.128219	8.934168	8.493348	32.01479	28.37733
20	12.44649	11.87344	12.06425	7.251771	7.276947	7.332643	17.04567	17.47637

$Ucp3^{-/-}$

0.935624	0.949781	1.04802	1.004632	1.078979	0.838956	1.104709	1.008585	1.007887
101.0319	39.13353	38.75561	38.72876	30.197	25.11249	22.22842	103.9443	116.6196
121.8813	30.05778	29.62343	30.07862	37.06537	35.77798	42.84337	105.4684	121.825
58.71539	19.74388	24.54459	20.86963	38.41683	37.49605	32.86925	44.48766	36.76684
27.75482	15.75058	15.38373	13.92237	19.46306	20.13546	17.72442	23.0602	24.05614
18.00509	5.693569	5.301253	4.725062	11.17343	10.25318	9.673245	11.40294	12.8112

0.983729
107.9835
99.15865
41.56604
22.13616
14.2445
