# Supplementary material for: UCP3 reciprocally controls CD4+ Th17 and Treg cell differentiation
Source: PLoS One. 2020 Nov 19;15(11):e0239713. doi: 10.1371/journal.pone.0239713 (PMC7676685; doi:10.1371/journal.pone.0239713)
Supplement: S8 File — (PDF) [file pone.0239713.s008.pdf]

$Ucp3^{+/+}$	$Ucp3^{-/-}$
1721	1812
1594	1815
1791	1801
