# Supplementary material for: UCP3 reciprocally controls CD4+ Th17 and Treg cell differentiation
Source: PLoS One. 2020 Nov 19;15(11):e0239713. doi: 10.1371/journal.pone.0239713 (PMC7676685; doi:10.1371/journal.pone.0239713)
Supplement: S9 File — (ZIP) [file pone.0239713.s009.zip › SS9D_File.pdf]

Th17	+ αIL-2	+ Isotype
15	62.3	11.9
13.3	37.6	13.9
14	40.5	13.4
