# Supplementary material for: UCP3 reciprocally controls CD4+ Th17 and Treg cell differentiation
Source: PLoS One. 2020 Nov 19;15(11):e0239713. doi: 10.1371/journal.pone.0239713 (PMC7676685; doi:10.1371/journal.pone.0239713)
Supplement: S9 File — (ZIP) [file pone.0239713.s009.zip › SS9E_File.pdf]

Th17	+ αIL-2	+ Isotype
377.105	9366.22	327.278
135.506	5393.41	166.212
288.5	7934.7	255.702
