# Supplementary material for: UCP3 reciprocally controls CD4+ Th17 and Treg cell differentiation
Source: PLoS One. 2020 Nov 19;15(11):e0239713. doi: 10.1371/journal.pone.0239713 (PMC7676685; doi:10.1371/journal.pone.0239713)
Supplement: S10 File — (PDF) [file pone.0239713.s010.pdf]

Combined OCR/ECAR Basal Th0 cells

UCP3+/+	UCP3-/-
4.942857	6.285714
4.948718	4.965517
4.777778	5.5625
3.846154	5.481482
3.444444	6.681818
4.025641	4.37931
3.28	5
4.352941	3.533333
3.658537	4.6
4.645161	4.034483
5.151515	6.894737
5.216216	5.571429
5	5.085714
3.775	5.961538
3.871795	7.619048
3.810811	4.916667
4.022222	6.291667
4.606061	4.137931
3.707317	5.24
5.137931	4.75
5.176471	6.095238
5.078948	5
5.242424	5.758621
4.027778	5.884615
3.948718	7.421052
5.1	5.217391
3.978261	6.478261
4.757576	4.275862
3.707317	5.541667
5.615385	4.821429
5.814815	6.892857
6.25	
8.65	5.875
7.045455	5.068965
5.884615	5.206897
7.25	4.935484
6.166667	4.551724
8.045455	4.2
5.333333	4.62069
4.846154	3.555556
5.925926	6.678571
6.826087	
8.95	5.677419
8.047619	5.357143
7.227273	5.076923
9	6.185185
6.517241	5.111111
8.952381	4.9375

5.833333	5.37037
5.708333	3.285714
6.75	7.24
7.761905	
10.38889	6.1
8.7	5.703704
8.333333	5
8.230769	6.576923
6.62069	5.428571
8.772727	4.967742
6.133333	5.888889
5.875	3.742857
8.736842	13.63636
8.692307	8.333333
13.8	8.166667
8.142858	5.347826
12.18182	7.333333
11.83333	7.4
9.4375	6
7.473684	4.75
6.428571	5.458333
9.846154	5.545455
10.8	12.58333
11	8.777778
15.55556	8.722222
10.54545	6.476191
11.72727	8.642858
15.45455	7.166667
11.92857	7.470588
9	6.047619
7.736842	6.272727
10.25	6.6
11.53333	15.36364
12.72727	9.705882
16.33333	9.055555
10.83333	7.35
13.9	9.785714
16	6.277778
14.08333	8
10.05882	6.55
8.470589	6.523809
11.25	7.105263
