# Supplementary figures and images for: UCP3 reciprocally controls CD4+ Th17 and Treg cell differentiation
Source: PLoS One. 2020 Nov 19;15(11):e0239713. doi: 10.1371/journal.pone.0239713 (PMC7676685; doi:10.1371/journal.pone.0239713)

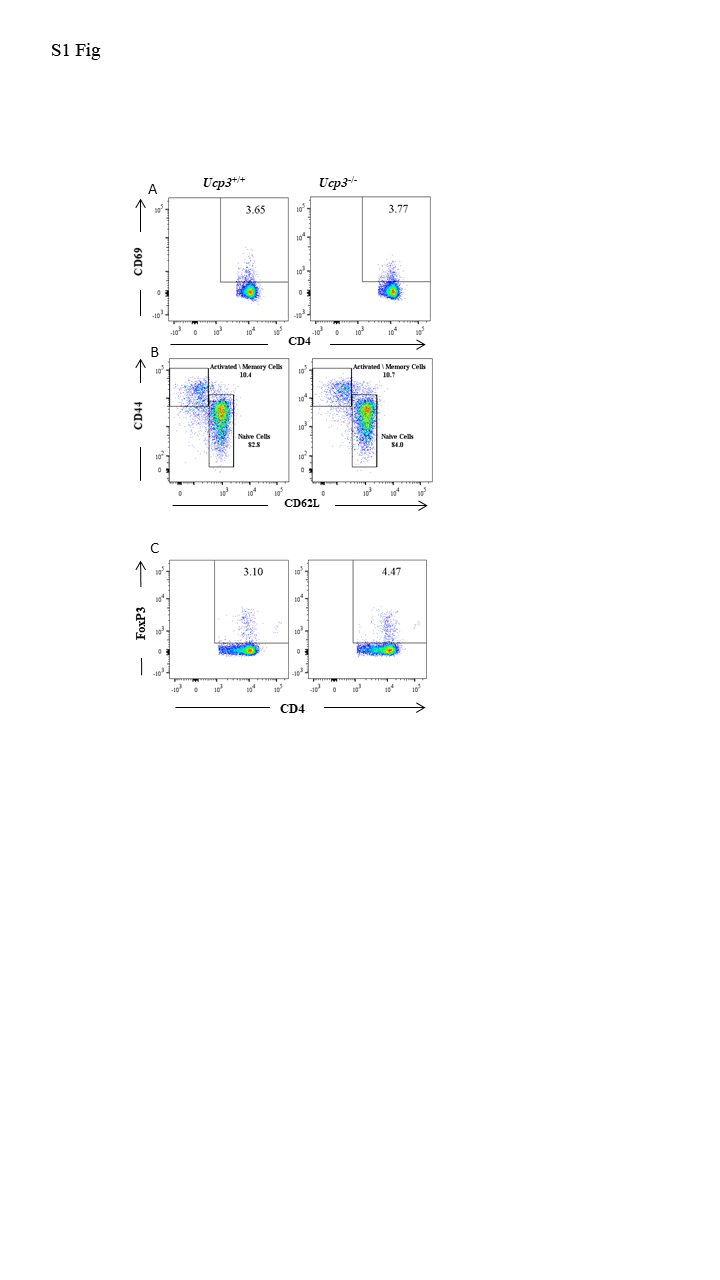

Supplement: S1 Fig — Primary CD4+ T cells were isolated from Ucp3+/+ and Ucp3-/- mice and the relative levels of early activated (CD69), naïve/memory CD4+ (CD44/CD62L), and Treg (FoxP3) subsets analysed. (A) CD69 expression of naive CD4+ T cells. (B) Frequency of naive and memory T cells. (C) FoxP3 and CD4 co-expression by CD4+ T cells. All data are presented as a percentage of the viable, CD4+ T cell population. Dot plots are representative of at least three different experiments. (TIF) [file pone.0239713.s011.tif]

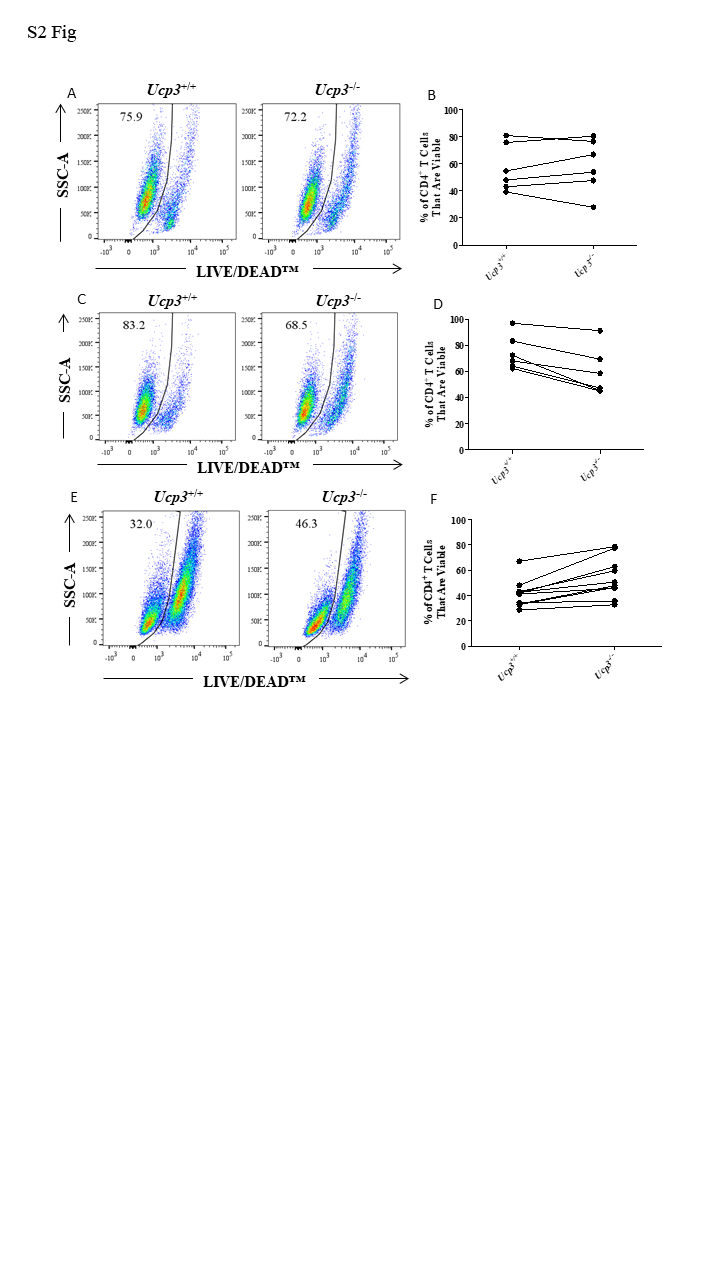

Supplement: S2 Fig — Differentiated Th subsets from Ucp3+/+ and Ucp3-/- mice shown in Fig 2 were analysed for viability through staining with LIVE/DEAD™ exclusion. (A&B) Representative and collated viability data for Ucp3+/+ and Ucp3-/- Th1 cells, (C&D) Th17 cells, (E&F) iTreg cells. Data are representative of six independent experiments. Data were analysed using a two-tailed, unpaired t test to quantify significance. Data are presented as a percentage of the total CD4+ T cell population. (TIF) [file pone.0239713.s012.tif]

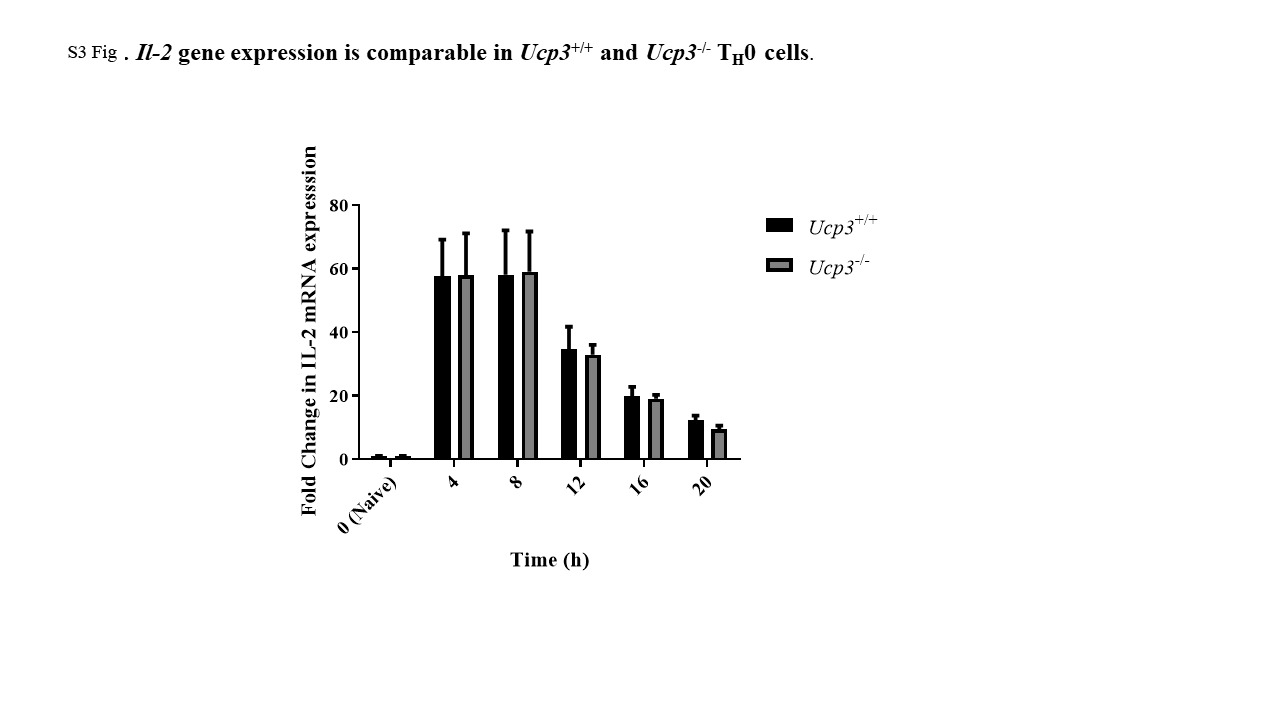

Supplement: S3 Fig — RT-PCR analysis of Il-2 gene expression in Th0 cells relative to naive T cells. Primary CD4+ T cells were isolated from a suspension of splenocytes and analysed by RT-PCR immediately or after activation under non polarising conditions (Th0) as described in the methods section (conditions were 1 ug/mL of anti-CD3 and 2 ug/mL of anti-CD28). RT-PCR was performed three times in triplicate. Data were analysed using a two-way ANOVA with a post hoc Bonferroni test to quantify significance where detected. (TIF) [file pone.0239713.s013.tif]

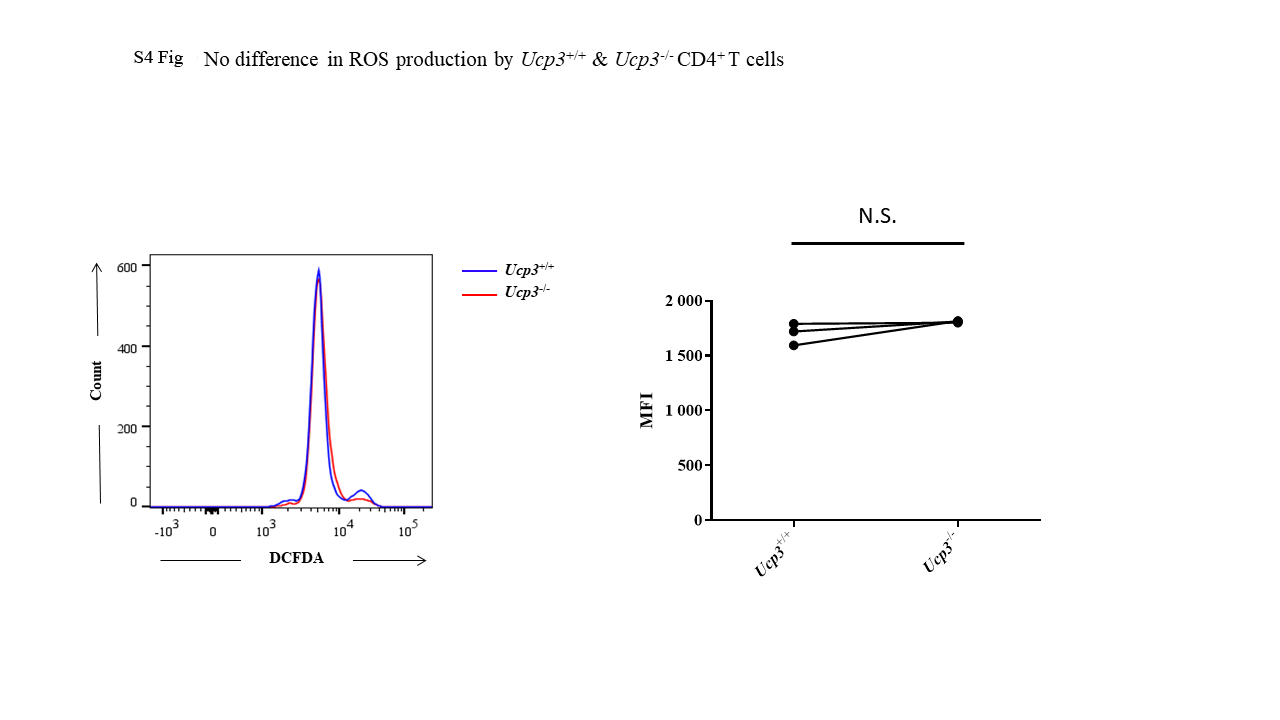

Supplement: S4 Fig — Primary CD4+ T cells were isolated from a suspension of splenocytes and stained with DCFDA before being analysed on a flow cytometer. An example of the primary data is given and the histogram represents three different experiments detecting ROS in viable CD4+ T cell population. All data were analysed using a two-tailed, unpaired t test. (TIF) [file pone.0239713.s014.tif]

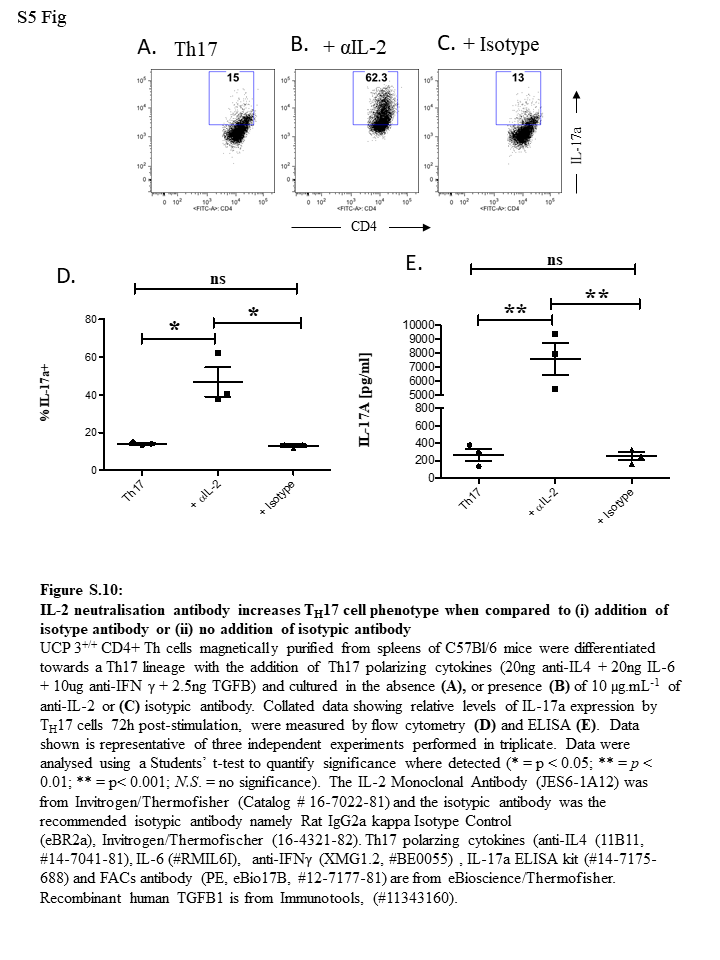

Supplement: S5 Fig — IL-2 neutralisation antibody increases TH17 cell phenotype when compared to (i) addition of isotype antibody or (ii) no addition of isotypic antibody UCP 3+/+ CD4+ Th cells magnetically purified from spleens of C57Bl/6 mice were differentiated towards a Th17 lineage with the addition of Th17 polarizing cytokines (20ng anti-IL4 + 20ng IL-6 + 10ug anti-IFN γ + 2.5ng TGFB) and cultured in the absence (A), or presence (B) of 10 μg.mL-1 of anti-IL-2 or (C) isotypic antibody. Collated data showing relative levels of IL-17a expression by TH17 cells 72h post-stimulation, were measured by flow cytometry (D) and ELISA (E). Data shown is representative of three independent experiments performed in triplicate. Data were analysed using a Students’ t-test to quantify significance where detected (* = p < 0.05; ** = p < 0.01; ** = p< 0.001; N.S. = no significance). The IL-2 Monoclonal Antibody (JES6-1A12) was from Invitrogen/Thermofisher (Catalog # 16-7022-81) and the isotypic antibody was the recommended isotypic antibody namely Rat IgG2a kappa Isotype Control (eBR2a), Invitrogen/Thermofischer (16-4321-82). Th17 polarzing cytokines (anti-IL4 (11B11, #14-7041-81), IL-6 (#RMIL6I), anti-IFNγ (XMG1.2, #BE0055), IL-17a ELISA kit (#14-7175-688) and FACs antibody (PE, eBio17B, #12-7177-81) are from eBioscience/Thermofisher. Recombinant human TGFB1 is from Immunotools, (#11343160). (TIF) [file pone.0239713.s015.tif]

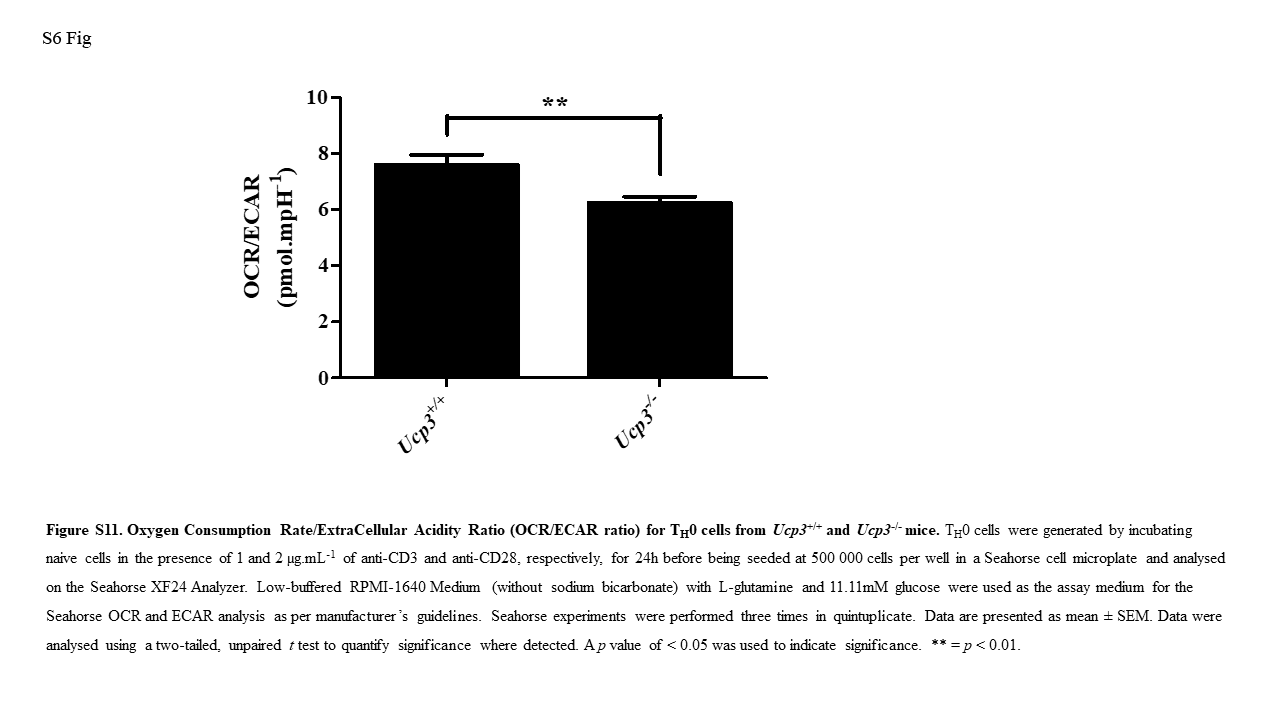

Supplement: S6 Fig — TH0 cells were generated by incubating naive cells in the presence of 1 and 2 μg.mL-1 of anti-CD3 and anti-CD28, respectively, for 24h before being seeded at 500 000 cells per well in a Seahorse cell microplate and analysed on the Seahorse XF24 Analyzer. Low-buffered RPMI-1640 Medium (without sodium bicarbonate) with L-glutamine and 11.11mM glucose were used as the assay medium for the Seahorse OCR and ECAR analysis as per manufacturer’s guidelines. Seahorse experiments were performed three times in quintuplicate. Data are presented as mean ± SEM. Data were analysed using a two-tailed, unpaired t test to quantify significance where detected. A p value of < 0.05 was used to indicate significance. ** = p < 0.01. (TIF) [file pone.0239713.s016.tif]
